# Targeting hypoxia-inducible factors with 32-134D safely and effectively treats diabetic eye disease in mice

**DOI:** 10.1172/JCI163290

**Published:** 2023-07-03

**Authors:** Jing Zhang, Deepti Sharma, Aumreetam Dinabandhu, Jaron Sanchez, Brooks Applewhite, Kathleen Jee, Monika Deshpande, Miguel Flores-Bellver, Ming-Wen Hu, Chuanyu Guo, Shaima Salman, Yousang Hwang, Nicole M. Anders, Michelle A. Rudek, Jiang Qian, M. Valeria Canto-Soler, Gregg L. Semenza, Silvia Montaner, Akrit Sodhi

**Affiliations:** 1 Wilmer Eye Institute, Johns Hopkins University School of Medicine, Baltimore, Maryland, USA.; 2 State Key Laboratory of Ophthalmology, Zhongshan Ophthalmic Center, Sun Yat-sen University, Guangzhou, Guangdong, China.; 3 Department of Oncology and Diagnostic Sciences, School of Dentistry, Greenebaum Comprehensive Cancer Center, University of Maryland, Baltimore, Maryland, USA.; 4 CellSight Ocular Stem Cell and Regeneration Research Program, Department of Ophthalmology, Sue Anschutz-Rodgers Eye Center, University of Colorado School of Medicine, Aurora, Colorado, USA.; 5 Armstrong Oxygen Biology Research Center; Vascular Program, Institute for Cell Engineering; Departments of Pediatrics, Medicine, Oncology, Radiation Oncology, Biological Chemistry, and Genetic Medicine, Johns Hopkins University School of Medicine, Baltimore, Maryland, USA.; 6 The Sidney Kimmel Comprehensive Cancer Center, Department of Oncology and the Division of Clinical Pharmacology at the School of Medicine, Johns Hopkins University, Baltimore, Maryland, USA.

**Keywords:** Ophthalmology, Therapeutics, Diabetes, Mouse models, Retinopathy

## Abstract

Many patients with diabetic eye disease respond inadequately to anti-VEGF therapies, implicating additional vasoactive mediators in its pathogenesis. We demonstrate that levels of angiogenic proteins regulated by HIF-1 and -2 remain elevated in the eyes of people with diabetes despite treatment with anti-VEGF therapy. Conversely, by inhibiting HIFs, we normalized the expression of multiple vasoactive mediators in mouse models of diabetic eye disease. Accumulation of HIFs and HIF-regulated vasoactive mediators in hyperglycemic animals was observed in the absence of tissue hypoxia, suggesting that targeting HIFs may be an effective early treatment for diabetic retinopathy. However, while the HIF inhibitor acriflavine prevented retinal vascular hyperpermeability in diabetic mice for several months following a single intraocular injection, accumulation of acriflavine in the retina resulted in retinal toxicity over time, raising concerns for its use in patients. Conversely, 32-134D, a recently developed HIF inhibitor structurally unrelated to acriflavine, was not toxic to the retina, yet effectively inhibited HIF accumulation and normalized HIF-regulated gene expression in mice and in human retinal organoids. Intraocular administration of 32-134D prevented retinal neovascularization and vascular hyperpermeability in mice. These results provide the foundation for clinical studies assessing 32-134D for the treatment of patients with diabetic eye disease.

## Graphical abstract

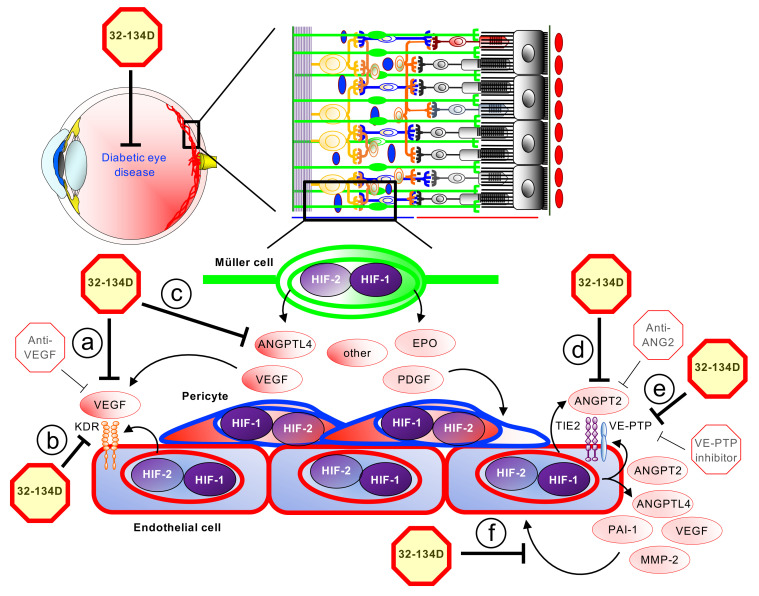

## Introduction

Identification of factors that trigger the development of nonproliferative diabetic retinopathy (NPDR) remains under debate; these factors include advanced glycosylation end products (AGEs), oxidative stress, and inflammation, all leading to damage of the retinal neurovascular unit (1, 2). Progressive damage to the retinal microvasculature results in the development of ischemia (3) and the increased expression of the angiogenic mediators that promote retinal neovascularization (NV) (4), heralding progression from NPDR to proliferative diabetic retinopathy (PDR). On the other hand, diabetic macular edema (DME) can occur prior to — or in the absence of — overt retinal ischemia. Despite seemingly disparate origins, therapies targeting a single vasoactive mediator, VEGF, have emerged as effective treatments for both PDR (5) and DME (6). Nonetheless, fewer than half of patients with PDR or DME respond adequately to current anti-VEGF therapies (7–10), prompting investigators to search for more effective approaches for treating diabetic eye disease.

One strategy for treating diabetic eye disease more effectively is to develop drugs targeting other vasoactive mediators that could be used alone or in combination with current anti-VEGF therapies. An extension of this strategy is to simultaneously target multiple vasoactive factors by inhibiting the transcriptional regulators that promote their expression. In this regard, a family of transcriptional activators, the hypoxia-inducible factors (HIFs), have emerged as the master regulators of the expression of hypoxia-regulated vasoactive mediators in retinal vascular disease (4). HIFs are heterodimeric proteins composed of an exquisitely oxygen-sensitive α subunit and a ubiquitously expressed β subunit (11). HIF-1α was the first HIF α subunit isoform to be identified (12), and its critical role has been subsequently demonstrated in retinal vascular disease. HIF-2α is closely related to HIF-1α and also activates hypoxia-inducible gene transcription, although its expression may be more limited.

Accumulation of both HIF-1α and HIF-2α has been reported in preclinical models of ischemic retinal disease (13–15) as well as in tissue from patients with ischemic retinopathies, including PDR (13–19). Conversely, there is a paucity of evidence supporting a role for HIFs in the development of early NPDR or DME. In the absence of ischemia, the expression of VEGF in patients with DME is believed to be independent of hypoxia or HIFs and has instead been attributed to other causes including AGEs, oxidative stress, and neuroinflammation, independent of HIF accumulation (1, 2). However, in addition to hypoxia, many inflammatory cytokines and growth factors previously implicated in diabetic eye disease (20) have also been shown to induce HIF activity in an oxygen-independent manner (21), often by mechanisms distinct from its canonical hypoxia-mediated regulation (22). Whether these oxygen-independent pathways influence HIFs and HIF-regulated gene expression in early NPDR or DME remains unclear.

While the most critical HIF-dependent secreted factor elaborated in diabetic eye disease is arguably VEGF, other genes regulated by HIFs also contribute to the pathogenesis of both PDR and DME (4). This includes the endothelial-expressed receptor for VEGFA, VEGF receptor 2 (VEGFR2 or KDR) (23), PDGFB (24), angiopoietin 2 (ANGPT2) and its downstream effector, vascular endothelial–protein tyrosine phosphatase (VE-PTP) (25), angiopoietin-like 4 (ANGPTL4) (15, 18, 26, 27), MMPs (19), and plasminogen activator inhibitor-1 (PAI-1) (13). Importantly, HIFs increase expression of these factors primarily under pathologic (e.g., retinal ischemia), not physiologic, conditions (28), making HIFs attractive targets for the treatment of diabetic eye disease. Nonetheless, development of a safe and effective therapy targeting both HIF-1 and HIF-2 has remained elusive. Here, we set out to evaluate the contribution of HIFs and HIF-regulated genes to both PDR and DME and to determine whether therapies targeting HIFs could be a safe and effective treatment for patients with diabetic eye disease.

## Results

### Vitreous expression of multiple HIF-dependent vasoactive mediators in PDR patients is unaffected by anti-VEGF therapy.

**Figure 1:**
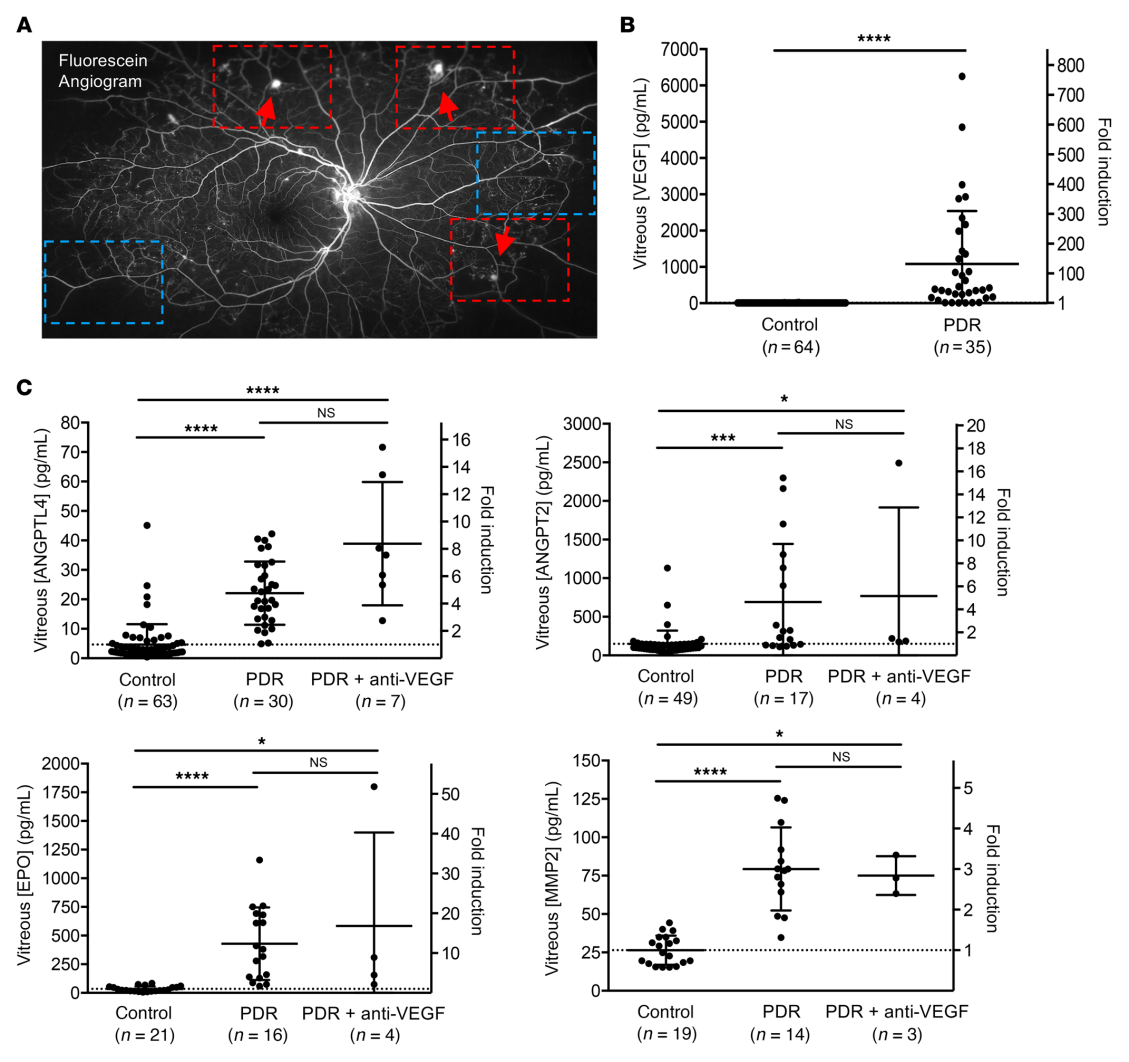
Vitreous expression of multiple HIF-dependent vasoactive mediators is unaffected by anti-VEGF therapy in PDR. (**A**) Fluorescein angiogram from a patient with PDR demonstrating areas of retinal nonperfusion with (red boxes) and without (blue boxes) retinal NV (red arrows). (**B**) Expression of VEGF in vitreous samples obtained from patients with PDR undergoing vitrectomy surgery who were treatment naive or who had not had anti-VEGF therapy for at least 12 weeks (PDR). (**C**) Expression of ANGPTL4, ANGPT2, EPO, and MMP2 in vitreous samples from patients with PDR who were treatment naive or who had not had anti-VEGF therapy for at least 12 weeks (PDR) or from patients with PDR who underwent recent treatment with anti-VEGF therapy within 6 weeks of sample collection (PDR+anti-VEGF). Vitreous samples from nondiabetic patients undergoing vitrectomy surgery for visually significant vitreous opacities (floaters) or epiretinal membranes were used as controls. Data are shown as mean ± SD. Statistical analyses were performed using 2-tailed Student’s *t* test (**B**) or 1-way ANOVA with Bonferroni’s multiple-comparison test (**C**). *P < 0.05; ***P < 0.001; ****P < 0.0001.

In diabetic retinopathy, sustained hyperglycemia damages retinal vessels and leads to progressive capillary dropout (2). If sufficient, this can cause profound retinal ischemia (3), leading to the accumulation of the transcription factors HIF-1 and HIF-2, and increased expression of VEGF (4). Increased VEGF, in turn, promotes the development of retinal NV (Figure 1A), the hallmark of PDR. To examine the expression of vasoactive mediators in patients with PDR, we collected vitreous samples from patients with PDR undergoing vitrectomy surgery who were treatment naive or who had not had anti-VEGF therapy for at least 12 weeks. Nondiabetic patients undergoing vitrectomy for visually significant vitreous opacities (floaters) or epiretinal membranes (which we previously reported do not influence the expression of vasoactive mediators despite the presence of macular edema; ref. 29) were used as controls. ELISA analysis for VEGF demonstrated an increase in expression of VEGF in PDR patients compared with nondiabetic controls (Figure 1B), as has previously been reported (30). Expression of other HIF-regulated vasoactive mediators previously implicated in PDR, such as ANGPTL4 (18, 27), ANGPT2 (31, 32), erythropoietin (EPO) (33), and MMP-2 (19, 34), were also increased in the vitreous of PDR patients and remained elevated despite treatment with anti-VEGF therapy within 6 weeks of sample collection (Figure 1C).

### Expression of multiple vasoactive mediators is normalized in the OIR model following treatment with the pharmacological HIF inhibitor digoxin.

**Figure 2:**
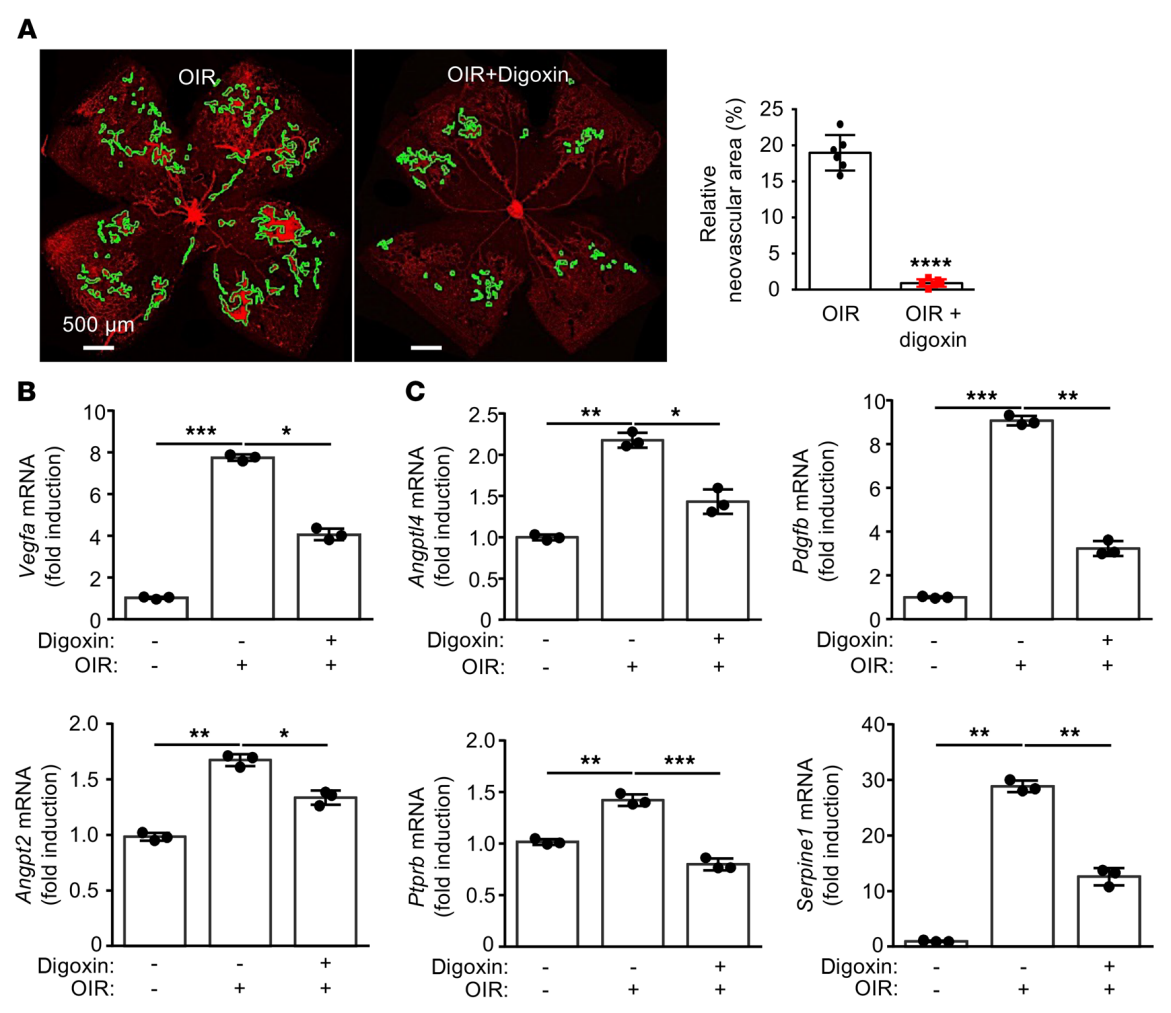
Modest inhibition of multiple HIF-dependent vasoactive mediators is sufficient for preventing retinal NV in the OIR mouse model. (**A**) Left: representative images of retinal NV in P17 OIR mice following a single i.p. injection with vehicle or digoxin (2 mg/kg) at P12. Right: quantitation of retinal NV at P17. (**B** and **C**) mRNA expression of *Vegf* (**B**) or other key HIF-regulated angiogenic mediators (**C**) expressed by Müller glial and/or vascular cells of OIR mice at P17 following treatment with vehicle or digoxin at P12 compared with control mice. *n* = 4–6 animals for each condition. Data are represented as mean ± SD. Statistical analyses were performed using 2-tailed Student’s *t* test (**A**) or 1-way ANOVA with Bonferroni’s multiple-comparison test (**B** and **C**). *P < 0.05; **P < 0.01; ***P < 0.001; ****P < 0.0001. Scale bars: 500 μm.

To characterize the contribution of these other HIF-regulated angiogenic factors to the promotion of ischemia-driven retinal NV in vivo, we used the classic oxygen-induced retinopathy (OIR) model characterized by Smith and colleagues (35). In stage 1 of this model, P7 pups are exposed to hyperoxia (75% O_2_) for 5 consecutive days (P7–P12), resulting in obliteration of the posterior retinal vasculature. In stage 2, pups (at P12) are returned to room air (21% O_2_). The resulting relative ischemia promotes the expression of angiogenic mediators that stimulate the development of retinal NV (peaking at P17; Figure 2A). Treatment of mice with the pharmacologic HIF inhibitor digoxin (36) prevented retinal NV in the OIR model (Figure 2A) by inhibiting the sequential accumulation of HIF-1α or HIF-2α (14). Interestingly, digoxin prevented retinal NV despite only modestly inhibiting the expression of VEGF (Figure 2B). Treatment with digoxin also resulted in a modest reduction of the expression of other vasoactive genes (Figure 2C), suggesting that the broad but modest inhibition of multiple HIF-regulated genes may be an effective strategy for the treatment of PDR.

### Aqueous expression of HIF-dependent vasoactive mediators is unaffected by anti-VEGF therapy in DME.

**Figure 3:**
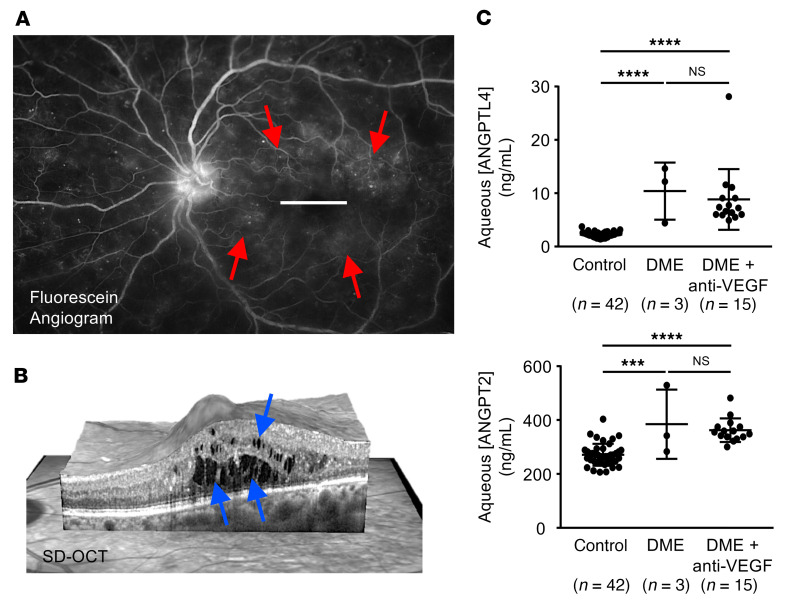
Aqueous expression of HIF-dependent vasoactive mediators is unaffected by anti-VEGF therapy in DME. (**A**) Fluorescein angiogram from a patient with NPDR and DME demonstrating diffuse leakage of fluid (red arrows) from retinal microaneurysms. (**B**) Spectral-domain optical coherence tomography (SD-OCT), providing a cross section of the macula (white line in **A**), demonstrating intraretinal fluid (blue arrows) in a patient with NPDR with DME. (**C**) Expression of ANGPTL4 and ANGPT2 in aqueous samples from patients with DME who are treatment naive or who had not been treated with anti-VEGF therapy for at least 12 weeks (DME) or from patients with DME following treatment with anti-VEGF therapy within 4 to 6 weeks of sample collection (DME+anti-VEGF). Aqueous fluid from nondiabetic patients obtained during routine cataract or vitrectomy surgery was used as control. Data are represented as mean ± SD. Statistical analyses were performed using 1-way ANOVA with Bonferroni’s multiple-comparison test. ***P < 0.001; ****P < 0.0001.

Patients with NPDR manifest injury to retinal vascular cells (i.e., endothelial cells and pericytes), resulting in the development of microaneurysms and vascular hyperpermeability (leakage of intravascular fluid; Figure 3A), often in the absence of overt retinal ischemia. When this fluid accumulates within the neurosensory retina, it results in the development of DME (Figure 3B). To examine the expression of vasoactive mediators in patients with DME, we collected aqueous samples from patients with NPDR undergoing intravitreal injections with anti-VEGF therapy for DME. Aqueous samples obtained from nondiabetic patients undergoing cataract surgery or vitrectomy surgery for vitreous opacities or epiretinal membranes were used as controls. We observed an increase in the expression of 2 key HIF-regulated angiogenic mediators, ANGPTL4 and ANGPT2, in the aqueous of patients with NPDR and DME who were treatment naive or who had not been treated with anti-VEGF therapy for at least 12 weeks; these levels remained unchanged following treatment with anti-VEGF therapy within 4 to 6 weeks of sample collection (Figure 3C).

### Increased expression of HIFs and HIF-regulated vasoactive mediators in a mouse model of sustained hyperglycemia in the absence of hypoxia.

**Figure 4:**
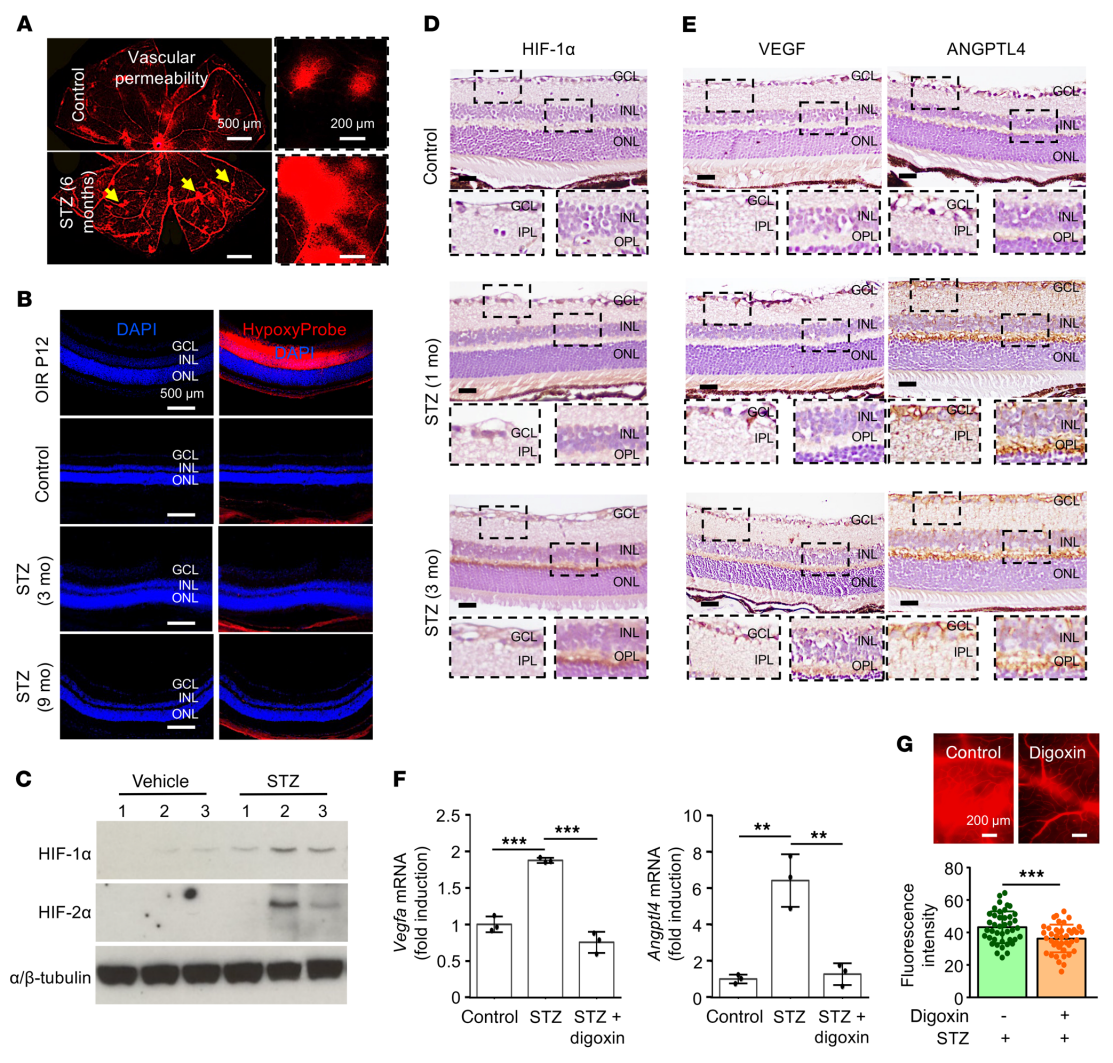
Increased expression of HIFs and HIF-regulated vasoactive mediators in a mouse model of sustained hyperglycemia in the absence of hypoxia. (**A**) Representative images of vascular hyperpermeability in STZ-induced diabetic mice that have been hyperglycemic for 6 months demonstrating leakage of Evans blue dye (yellow arrows) from retinal vessels, similar to the leakage observed in the fluorescein angiogram in patients with DME. (**B**) Hypoxia (as measured by Hypoxyprobe) in retinal tissue in P12 OIR mice (positive control) compared with that in STZ-induced diabetic mice that have been hyperglycemic for 0 (control), 3, or 9 months. (**C**) Western blot demonstrating accumulation of HIF-1α or HIF-2α protein in retinal tissue in STZ-induced diabetic mice that have been hyperglycemic for 6 months; α/β-tubulin was used as a loading control. (**D** and **E**) Representative images demonstrating expression of HIF-1α (**D**), VEGF, and ANGPTL4 (**E**) by immunohistochemistry in STZ mice that were hyperglycemic for 1 month or 3 months. (**F**) *Vegf* and *Angptl4* mRNA expression in STZ mice treated with digoxin. (**G**) Vascular hyperpermeability after digoxin treatment in STZ mice that were hyperglycemic for 6 months. *n* = 4–6 animals for each condition. IPL, inner plexiform layer; OPL, outer plexiform layer. Data are represented as mean ± SD. Statistical analyses were performed by 1-way ANOVA with Bonferroni’s multiple-comparison test (**F**) or 2-tailed Student’s t test (**G**). **P < 0.01; ***P < 0.001. Scale bars: 500 μm (**A** and **B**); 200 μm (**D**, **E**, and **G**). Original magnification, ×20 (**D** and **E**).

To examine the contribution of HIFs to the development of DME, we used the streptozotocin-induced (STZ-induced) diabetic mouse model. In this model, STZ is used to injure pancreatic β islet cells, leading to reduced insulin secretion and sustained hyperglycemia and secondary injury of the retinal microvasculature (37, 38). STZ mice with sustained hyperglycemia (blood glucose level >250 mg/dL) for 6 months demonstrated increased vascular permeability (Figure 4A), similar to that observed in patients with DME (see Figure 3A). Increased hypoxia (measured using the hypoxia-sensitive pimonidazole Hypoxyprobe) was not detected in retinal tissue from STZ mice despite up to 9 months of sustained hyperglycemia (Figure 4B). Nonetheless, accumulation of HIF-1α and HIF-2α protein was observed in immunoblot assays of neurosensory retina lysates from STZ mice that were hyperglycemic for 6 months (Figure 4C). Accumulation of HIF-1α protein could be detected by immunohistochemistry in retinal tissue from STZ mice after as little as 1 month of sustained hyperglycemia (Figure 4D). This correlated with an increase in the mRNA (Supplemental Figure 1; supplemental material available online with this article; https://doi.org/10.1172/JCI163290DS1) and protein (Figure 4E) expression of HIF-regulated vasoactive mediators compared with that of control mice. Treatment of STZ mice with the pharmacological HIF-inhibitor digoxin resulted in a decrease in *Vegf* and *Angptl4* mRNA expression to levels similar to those in nondiabetic mice (Figure 4F) and decreased vascular permeability (Figure 4G), suggesting that the broad inhibition of multiple HIF-regulated genes may be an effective strategy for the treatment of DME.

### The pharmacologic HIF inhibitor acriflavine accumulates in the neurosensory retina and inhibits retinal function over time.

**Figure 5:**
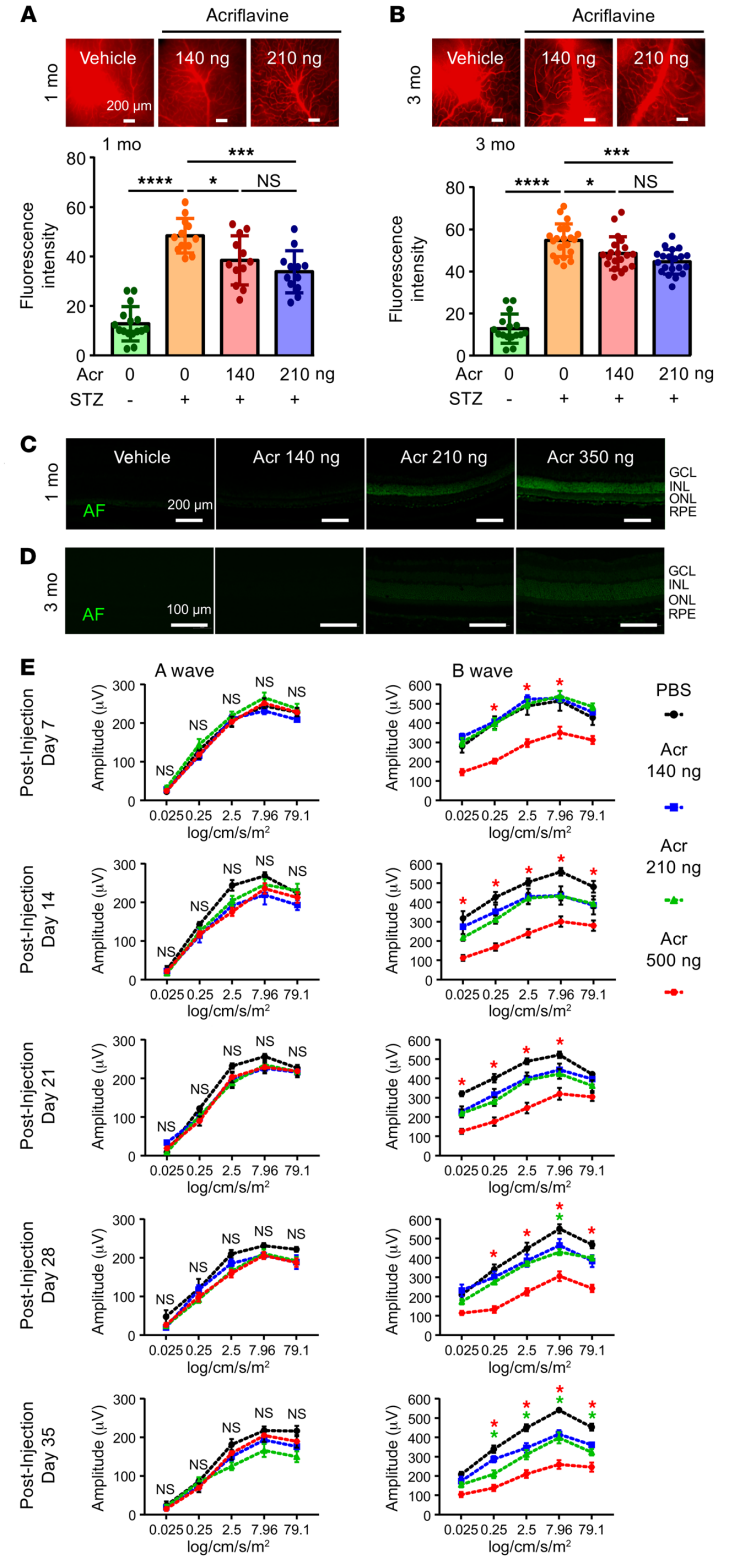
Acriflavine accumulates in the neurosensory retina and inhibits retinal function. (**A** and **B**) Representative images (above) and quantitation (below) of vascular permeability in STZ mice that were hyperglycemic for 6 months and treated with a single intraocular injection with PBS (vehicle) or acriflavine (140 ng or 210 ng) 1 month (**A**) or 3 months (**B**) prior to sacrificing animals. Vascular hyperpermeability was assessed by measuring Evans blue dye leakage. (**C** and **D**) Intrinsic autofluorescence of acriflavine accumulating in the neurosensory retina 1 month (**C**) or 3 months (**D**) following a single intraocular injection at the stated doses. (**E**) ERG of C57BL/6 mice 7 to 35 days following a single intraocular administration with PBS (vehicle) or acriflavine (Acr) at the stated doses. *n* = 5 animals for each group. RPE, retinal pigment epithelium. Data are represented as mean ± SD (**A** and **B**) or mean ± SEM (**E**). Statistical analyses were performed using 1-way ANOVA (**A** and **B**) or 2-way ANOVA (**E**) with Bonferroni’s multiple-comparison test. *P < 0.05; ***P < 0.001; ****P < 0.0001. Scale bars: 200 μm (**A** and **C**); 100 μm (**D**). Scale bars: 100 μm.

While digoxin is an effective research tool for examining the contribution of HIFs to retinal disease, use of this cardiac glycoside at effective concentrations to inhibit HIF is unlikely to be well tolerated by patients given its complex pharmacokinetic profile and narrow therapeutic index (39). Conversely, preclinical studies of a second pharmacologic HIF inhibitor, acriflavine (40), for the treatment of ocular vascular disease have been promising (41). We therefore explored the use of acriflavine for the treatment of vascular hyperpermeability in STZ mice. To this end, we administered a single intravitreal dose (140 or 210 ng) of acriflavine to STZ mice that were hyperglycemic for 9 months and observed a reduction in vascular hyperpermeability 1 month following treatment (Figure 5A), consistent with prior reports demonstrating its sustained efficacy for the treatment of other ocular neovascular diseases (41). Remarkably, this effect was sustained 3 months after a single intravitreal injection (Figure 5B).

The prolonged activity of acriflavine following a single injection suggested that there may be an ocular tissue depot of acriflavine following its administration and that this may facilitate its release over time. Consistent with this hypothesis, we took advantage of the fact that acriflavine is a DNA-intercalating fluorophore and observed a dose-dependent accumulation of acriflavine in the neurosensory retina 1 and 3 months following a single intraocular administration (Figure 5, C and D). These results help explain how a single intraocular injection with acriflavine can have a sustained effect on vascular permeability in mice 3 months after the injection.

While therapeutically advantageous, the accumulation of acriflavine in the retina prompted us to look more closely for evidence of retinal toxicity. At therapeutic doses (140 and 210 ng), we did not observe evidence of retinal toxicity as assessed using electroretinograms (ERGs) 1 week after administration (Figure 5E), consistent with prior reports (41). However, over time, we observed evidence of increasing retinal toxicity with flattening of the ERG b wave as early as 2 weeks following intraocular administration of 140 or 210 ng, which reached statistical significance by 4 weeks for 210 ng and continued to progress at 5 weeks (Figure 5E). The 140 ng dose of acriflavine was not toxic within the 5-week time frame of the study. Nonetheless, these results raised concerns about the long-term safety of acriflavine for the treatment of ocular disease.

### Ocular administration of acriflavine promotes retinal cell death over time.

**Figure 6:**
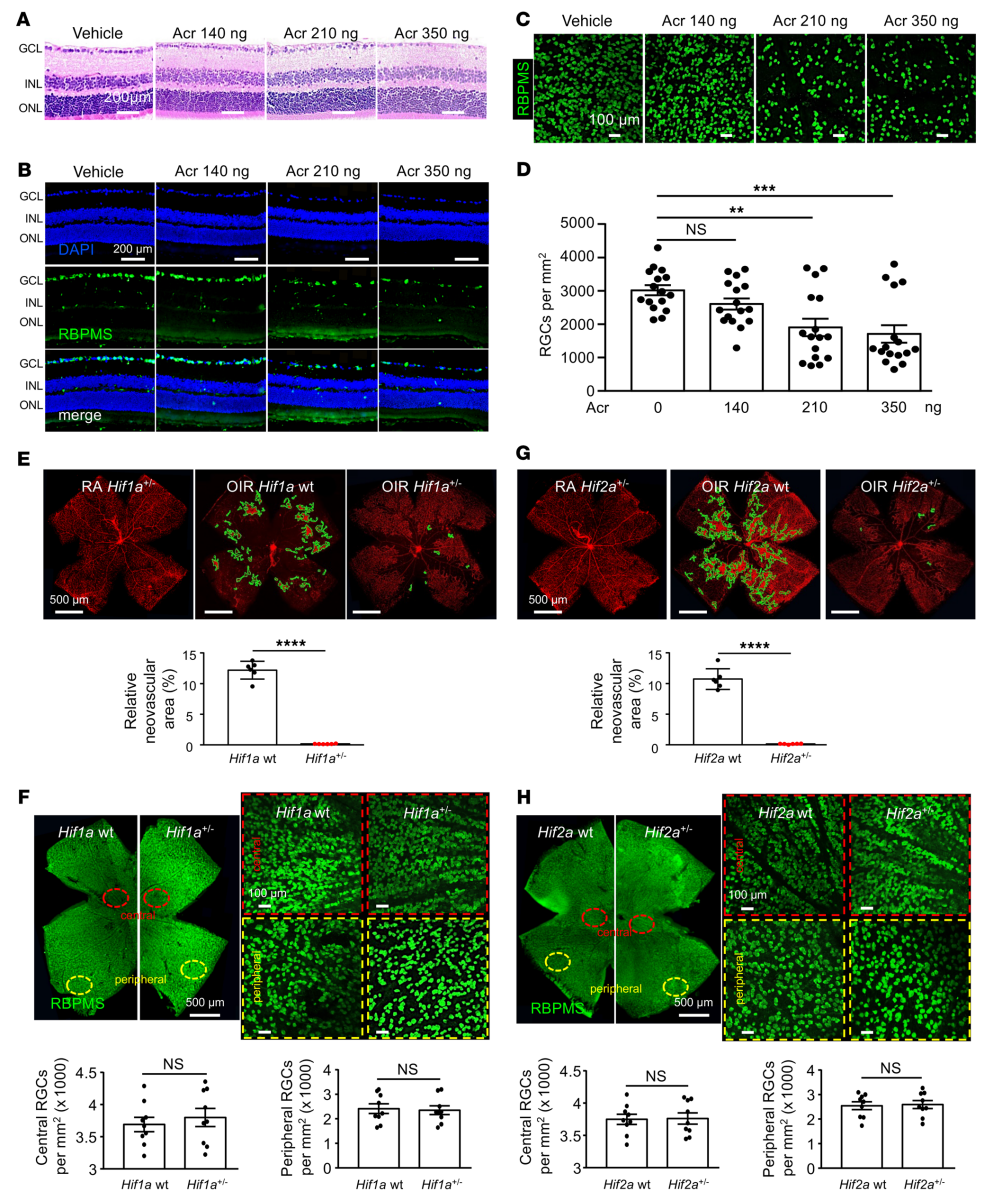
Acriflavine induces retinal cell death. (**A**) Representative images of H&E staining of retinal sections 35 days following a single intraocular injection with acriflavine at the stated doses. (B and C) Representative images of RBPMS staining of RGCs in retinal cross sections (**B**) and flat mounts (**C**) 35 days following a single intraocular injection with Acr at the stated doses. (**D**) Quantitation of RGCs in **C**. (**E** and **G**) Above: representative images of retinal NV in P17 non-OIR (RA) or OIR *Hif1a^+/–^* (**E**) or *Hif2a^+/–^* (**G**) mice compared with their WT littermates. Below: quantitation of retinal NV at P17. (F and **H**) Above: representative image of RBPMS staining of RGCs in central and peripheral retinal flat mounts of 6-week-old *Hif1a^+/–^* (**F**) or *Hif2a^+/–^* (**H**) mice compared with their WT littermates. Below: quantitation of RGCs in central and peripheral retinal flat mounts. *n* = 6 animals. Data are represented as mean ± SD. Statistical analyses were performed using 1-way ANOVA with Bonferroni’s multiple-comparison test (**D**) or 2-tailed Student’s *t* test (**E**–**H**). **P < 0.01; ***P < 0.001; ****P < 0.0001. Scale bars: 100 μm (**C**, **F** and **H**); 200 μm (**A** and **B**); 500 μm (**E**–**H**).

To further assess the retinal toxicity of acriflavine, we examined retinal sections 5 weeks after a single intraocular injection of acriflavine (140, 210, or 350 ng) and observed thinning of the ganglion cell layer (GCL) at 210 and 350 ng (Figure 6A). Immunofluorescence for the retinal ganglion cell (RGC) marker RBPMS demonstrated drop out of RGCs 5 weeks following treatment with acriflavine (Figure 6B). Quantitation of RGCs on retinal flat mounts demonstrated a dose-dependent decrease in RGCs that reached statistical significance at 210 ng (Figure 6, C and D). Collectively, these results indicate that acriflavine has a narrow therapeutic window in mice, raising concern about the safety of its use in patients with a chronic ocular disease.

To assess whether acriflavine’s retinal toxicity was a consequence of its on-target inhibition of HIF-1, we examined 6-week-old adult mice that were heterozygous for a knockout allele at the *Hif1a* locus (*Hif1a^+/–^*) (42). Basal levels of HIF-1α are relatively normal in *Hif1a^+/–^* mice, whereas in response to ischemia, HIF-1α expression is largely unchanged in *Hif1a^+/–^* mice, but significantly increased in WT littermate controls (43). Consequently, *Hif1a^+/–^* mice exhibited markedly decreased pathological angiogenesis in response to ischemia, as assessed by the OIR model (Figure 6E). ERGs (Supplemental Figure 2A), retinal histology (Supplemental Figure 2B), and quantitation of RGC number (Figure 6F) in 6-week-old adult *Hif1a^+/–^* mice were identical to those of their WT littermate controls. Similar results were obtained using 6-week-old mice that were heterozygous for a knockout allele at the *Hif2a* locus (*Hif2a^+/–^*) (Figure 6, G and H and Supplemental Figure 2C and D). Collectively, these results suggest that acriflavine toxicity is due to off-target effects, independent of its inhibition of HIF-1 or HIF-2.

### HIF inhibitor 32-134D effectively inhibits HIF accumulation and expression of HIF-regulated genes in retinal Müller cells.

**Figure 7:**
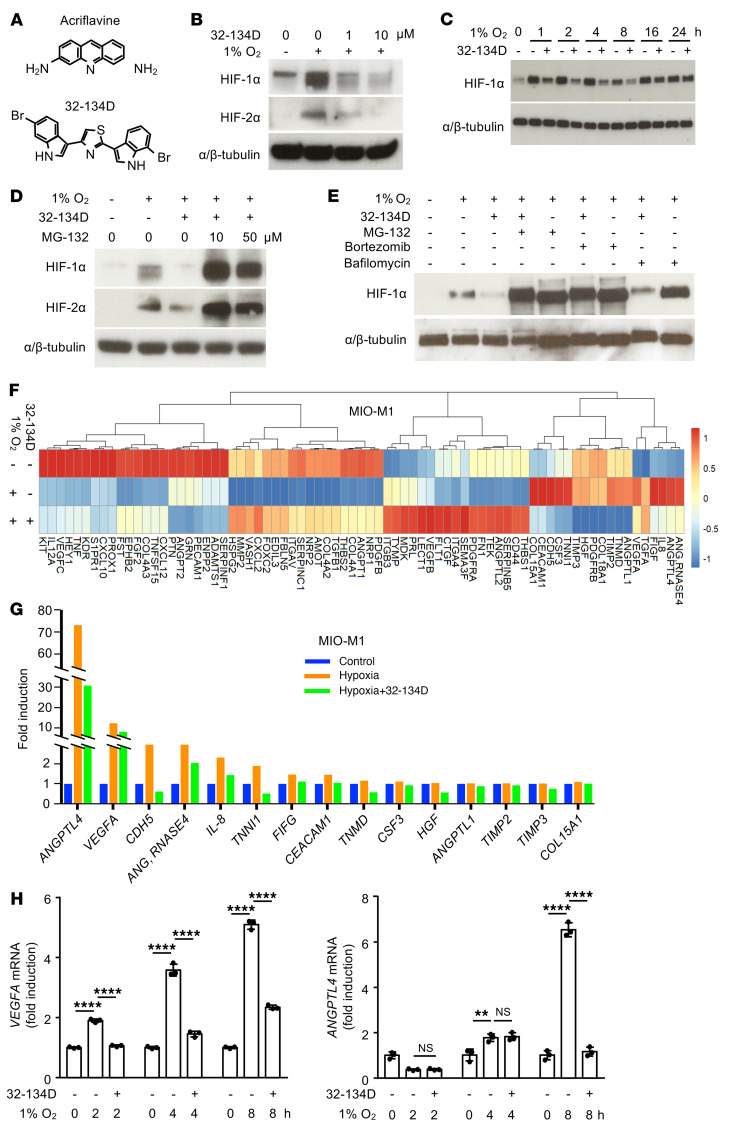
HIF inhibitor 32-134D effectively inhibits HIF accumulation and expression of HIF-regulated genes in MIO-M1 cells. (**A**) Chemical structures of acriflavine (above) and 32-134D (below). (**B**) Western blot demonstrating inhibition of HIF-1α and HIF-2α accumulation by 32-134D (1 or 10 μM) in MIO-M1 cells cultured in hypoxia (1% O_2_) for 4 hours. (**C**) Western blot demonstrating inhibition of HIF-1α accumulation by 32-134D (10 μM) in MIO-M1 cells cultured in hypoxia for 1 to 24 hours. (**D**) Effect of MG-132 on 32-134D inhibition of HIF-1α and HIF-2α accumulation in MIO-M1 cells cultured in hypoxia for 4 hours. (**E**) Effect of MG-132 (10 μM), bortezomib (10 μM), or bafilomycin (10 nM) on 32-134D inhibition of HIF-1α accumulation in MIO-M1 cells cultured in hypoxia for 4 hours. (**F**) Clustering analysis of angiogenesis array by qPCR screening for MIO-M1 cells cultured in the absence or presence of 32-134D and 1% O_2_ (hypoxia) or 20% O_2_ (normoxia) for 8 hours. Expression values were scaled in row direction, and “complete” was the default method in clustering method. (**G**) Multiple angiogenic genes were upregulated in cells cultured in hypoxia compared with control in angiogenesis array. (**H**) *VEGF* and *ANGPTL4* mRNA expression in MIO-M1 cells cultured in hypoxia (at indicated times) treated with vehicle or 32-134D (10 μM). Data are represented as mean ± SD. Statistical analyses were performed using 2-way ANOVA with Bonferroni’s multiple-comparison test. **P < 0.01; ****P < 0.0001.

The narrow therapeutic window for acriflavine prompted us to look for an alternative HIF inhibitor with a more desirable therapeutic profile. To this end, we recently interrogated the NCI CellMiner database (containing expression data on 25,698 mRNAs in 60 human cancer cell lines exposed to 21,770 chemical compounds) for small molecule inhibitors that produced changes in gene expression that were highly correlated with those induced by acriflavine, but that were structurally unrelated to acriflavine. Compounds that satisfied these criteria were further analyzed for their ability to inhibit HIF-dependent gene expression in a cell-based reporter assay (44). Using this approach, a hit, designated 11-88, was identified, and 224 analogs were synthesized. Among them, 27 compounds inhibited HIF transcriptional activity with IC_50_ of less than 4 μM, including the lead compound, a small molecule (<500 Daltons), 4-(6-bromo-1H-indol-3-yl)-2-(7-bromo-1H-indol-3-yl)thiazole, designated 32-134D (44), which is structurally unrelated to acriflavine (Figure 7A).

It has previously been reported that the cells in the inner nuclear layer (INL) of the retina that express VEGF in response to hypoxia/ischemia are Müller glial cells (15). Treatment of the immortalized human Müller cell line, MIO-M1, with as little as 1 μM of 32-134D resulted in decreased HIF-1α and HIF-2α protein accumulation in response to hypoxia (Figure 7B). The effects of 32-134D on HIF-1α accumulation persisted for up to 16 hours after treatment of cultured cells (Figure 7C). Pretreatment with the proteasome inhibitor MG-132 prevented the ability of 32-134D to block HIF-1α and HIF-2α protein accumulation (Figure 7D), indicating that 32-134D induced proteasomal degradation of HIF-1α subunits in MIO-M1 cells, as previously described for Hep3B human hepatocellular carcinoma cells (44). Similar results were obtained for HIF-2α (Figure 7D). These results were corroborated using another proteasome inhibitor, bortezomib (Figure 7E). Conversely, bafilomycin, an inhibitor of lysosomal acidification, did not prevent 32-134D from inhibiting HIF-1α accumulation in response to hypoxia (Figure 7E). Collectively, these results demonstrate that 32-134D promotes the proteasome-dependent degradation of both HIF-1α and HIF-2α protein despite the presence of hypoxia.

To assess the effects of 32-134D on angiogenic gene expression, we used an mRNA angiogenesis array and compared the angiogenic gene expression profile of MIO-M1 cells cultured in hypoxia in the absence or presence of 32-134D; cells cultured in normoxia were used as a control (Figure 7F). Multiple angiogenic genes were upregulated in cells cultured in hypoxia compared with control, most notably *ANGPTL4* and *VEGF* (Figure 7G); these results were confirmed by quantitative PCR (qPCR) (Figure 7H). While the levels of the mRNA transcripts of these angiogenic genes remained modestly elevated in hypoxic cells cultured in the presence of 32-134D compared with controls, they were substantially reduced compared with cells cultured in hypoxia. Indeed, treatment of hypoxic MIO-M1 cells with 32-134D resulted in a reduction in the Euclidean distance between control and hypoxic cell angiogenic gene expression from 73 to 31 (a reduction of 58%), suggesting that 32-134D can help normalize the expression of HIF-regulated angiogenic genes in hypoxic Müller cells.

### 32-134D inhibits the expression of HIF-regulated genes in vascular endothelial cells.

**Figure 8:**
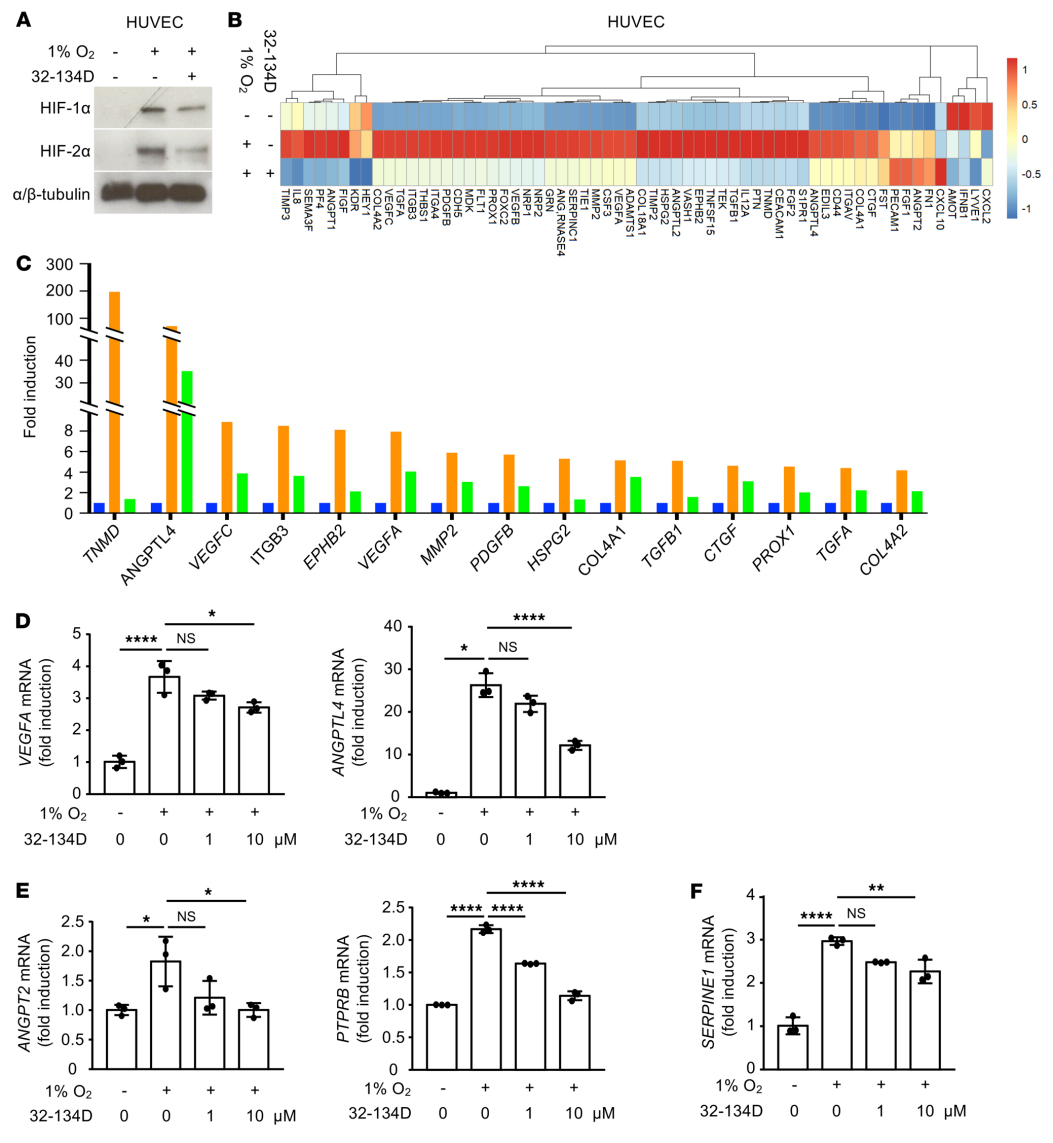
HIF inhibitor 32-134D effectively inhibits HIF accumulation and expression of HIF-regulated genes in endothelial cells. (**A**) Western blot demonstrating inhibition of HIF-1α and HIF-2α accumulation by 32-134D (10 μM) in HUVECs cultured in hypoxia for 4 hours. (**B**) Clustering analysis of angiogenesis array by qPCR screening for HUVECs cultured in the absence or presence of 32-134D and 1% O_2_ (hypoxia) or 20% O_2_ (normoxia) for 16 hours. Expression values were scaled in row direction, and “complete” was the default method in clustering method. (**C**) Multiple angiogenic genes were upregulated in cells cultured in hypoxia compared with control in angiogenesis array. (**D**–**F**) VEGF, ANGPTL4 (**D**), ANGPT2, PTPRB (**E**), and SERPINE1 (**F**) mRNA expression in HUVECs cultured in hypoxia for 16 hours and treated with vehicle or 32-134D (10 μM). Data are represented as mean ± SD. Statistical analyses were performed using 1-way ANOVA test with Bonferroni’s multiple-comparison test. *P < 0.05; **P < 0.01; ****P < 0.0001.

Vascular endothelial cells also secrete angiogenic mediators that contribute to the progression of diabetic eye disease (13). Exposure of HUVECs to hypoxia induced both HIF-1α and HIF-2α protein accumulation, which was also inhibited by 32-134D (Figure 8A). To assess the effects of 32-134D on angiogenic gene expression in HUVECs, we again used the mRNA angiogenesis array and compared HUVECs cultured in hypoxia in the absence or presence of 32-134D (Figure 8B). Over a dozen angiogenic genes were upregulated in HUVECs cultured in hypoxia compared with control, including *ANGPTL4* and *VEGF* (Figure 8C), similar to what was observed with MIO-M1 cells. The results for *VEGF* and *ANGPTL4* were confirmed by qPCR (Figure 8D).

ANGPT2 and VE-PTP, two emerging targets for the treatment of diabetic eye disease, are both expressed specifically by vascular cells (45, 46). Treatment of HUVECs with hypoxia resulted in increased expression of *ANGPT2* and *PTPRB* mRNA, which was inhibited by 32-134D (Figure 8E). Similar results were obtained for the recently identified HIF-2–dependent vascular cell–specific paracrine angiogenic mediator PAI-1 (13) (Figure 8F). Similarly to what occurs with MIO-M1 cells, treatment of hypoxic HUVECs with 32-134D resulted in a reduction in the Euclidean distance between control and hypoxic cell angiogenic gene expression from 209 to 35 (a reduction of 83%). Collectively, these results demonstrate that 32-134D can effectively inhibit the hypoxia-induced accumulation of both HIF-1α and HIF-2α and the expression of the key angiogenic genes they regulate in multiple cell types.

### 32-134D inhibits HIF accumulation and expression of HIF-regulated genes in human-inducible pluripotent stem cell–derived 3D retinal organoids.

**Figure 9:**
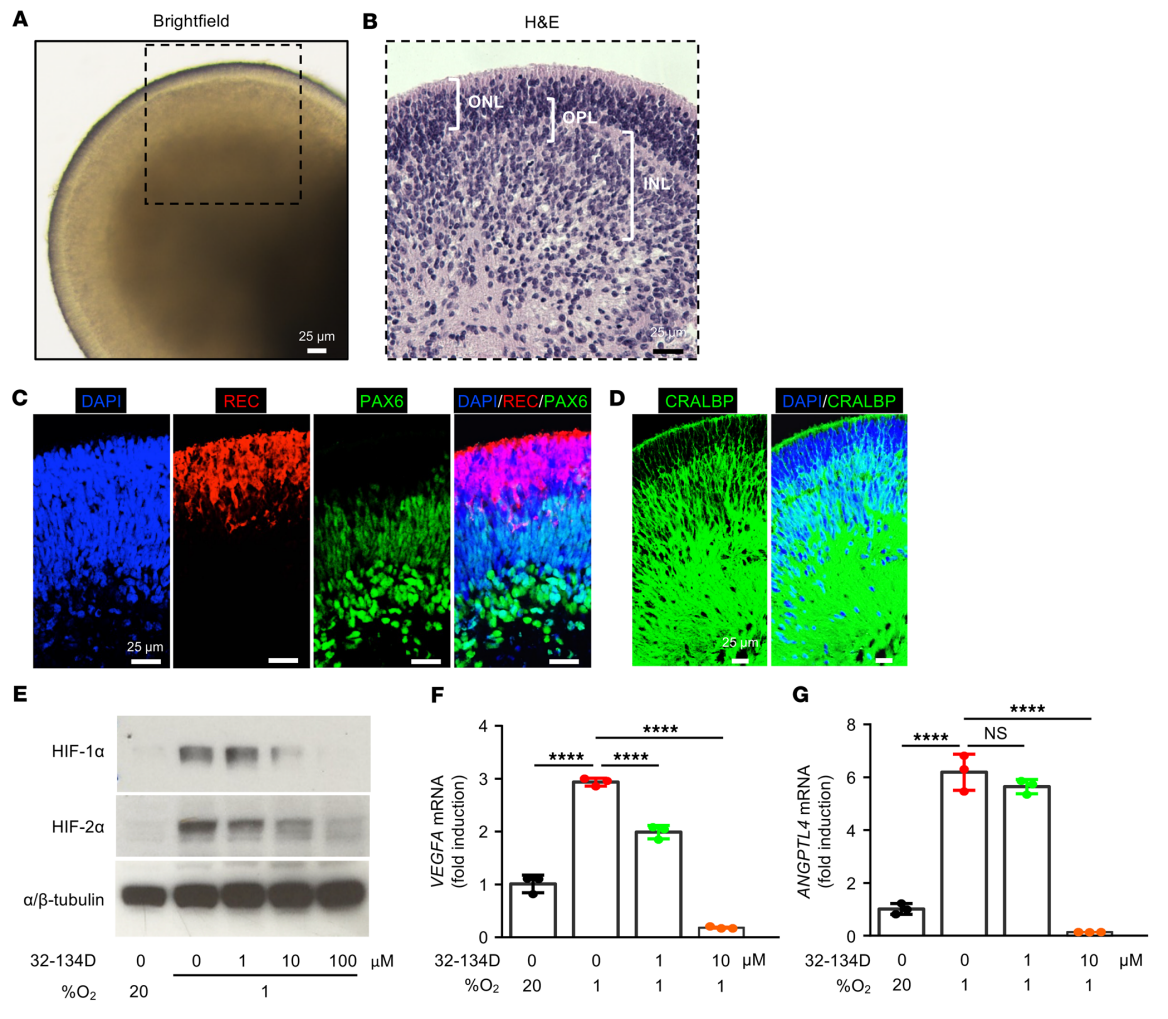
32-134D inhibits HIF accumulation and expression of HIF-regulated genes in hiPSC-derived 3D retinal organoids. (**A**) Representative bright-field image of D120 retinal organoid derived from hiPSCs. (**B**) H&E staining of D120 hiPSC-derived retinal organoid. (**C** and **D**) Representative immunofluorescence images of D120 hiPSC-derived retinal organoid demonstrating staining for Pax6 and recoverin (REC) (**C**) and CRALBP (**D**). (**E**) Inhibition of HIF-1α and HIF-2α accumulation in D120 hiPSC-derived retinal organoids cultured in 1% O_2_ for 12 hours and treated with 32-134D at indicated doses. (**F** and **G**) *VEGF* (**F**) and *ANGPTL4* (**G**) mRNA expression D120 hiPSC-derived retinal organoids cultured in 1% O_2_ for 12 hours and treated with 32-134D at indicated doses. *n* = 6–10 retinal organoids per condition. Data are represented as mean ± SD. Statistical analyses were performed using 1-way ANOVA with Bonferroni’s multiple-comparison test. ****P < 0.0001. Scale bars: 25 μm.

To assess the therapeutic potential of 32-134D for diabetic patients, we next treated human induced pluripotent stem cell–derived (hiPSC-derived) 3D retinal organoids. It has been previously reported that retinal organoids cultured under hypoxic conditions (1% O_2_) behave similarly to ischemic human retinal tissue with increased accumulation of both HIF-1α and HIF-2α (14). By 120 days of differentiation (D120), the inner and outer retinal layers of retinal organoids are clearly defined (Figure 9, A and B) and contain the precursors of the major retinal cell types, including outer retina photoreceptors (expressing recoverin), few newly differentiating bipolar cell precursors (lacking expression of recoverin and Pax6), and amacrine cells (expressing high levels of Pax6) as well as Müller cells (expressing CRALBP; Figure 9, C and D). Treatment of D120 retinal organoids cultured in 1% O_2_ with 32-134D effectively prevented accumulation of HIF-1α and HIF-2α protein (Figure 9E) and expression of HIF-regulated vasoactive mediators (Figure 9, F and G).

### Systemic administration of a well-tolerated dose of 32-134D inhibits HIF and HIF-regulated gene expression and effectively treats retinal vascular disease in mice.

**Figure 10:**
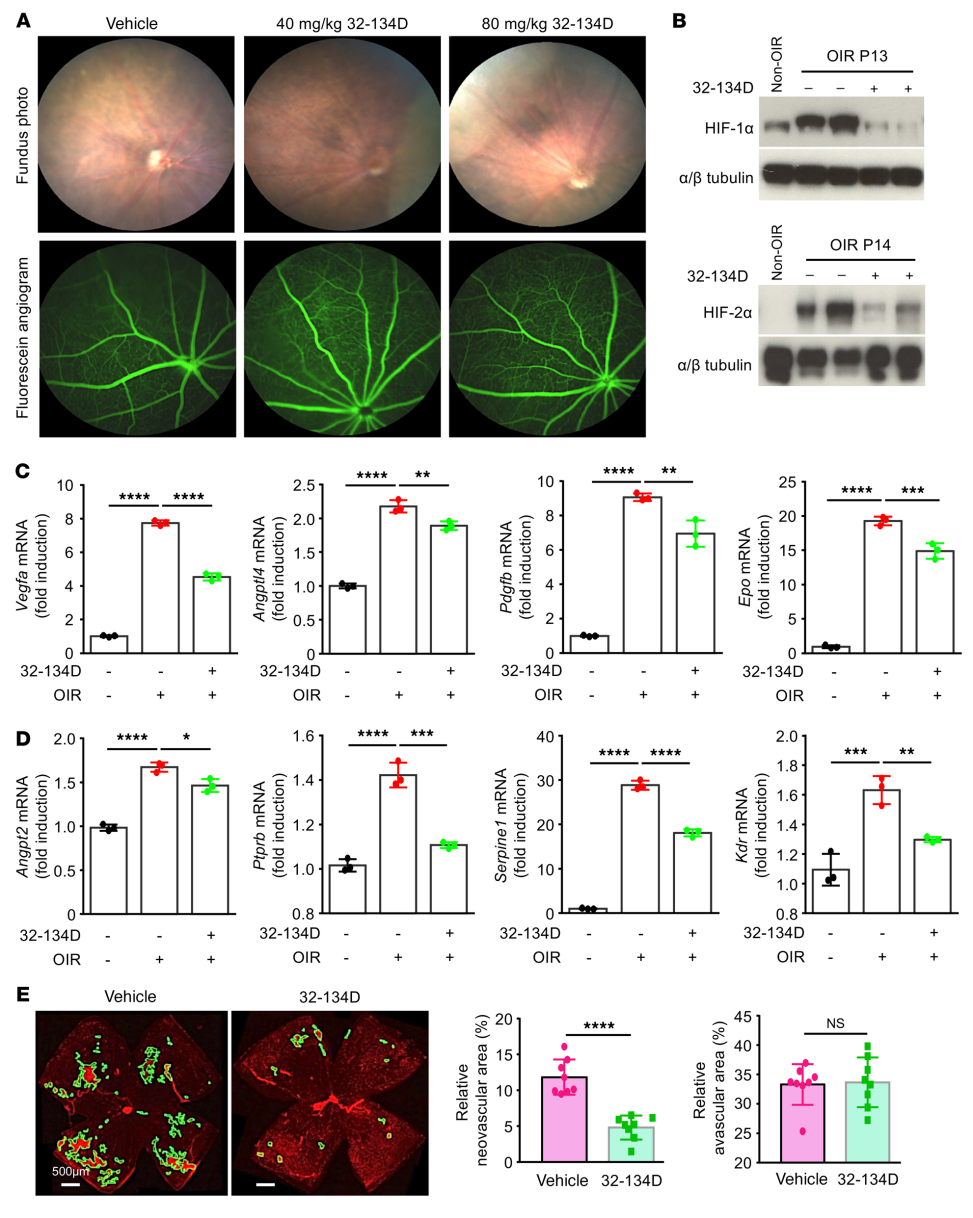
Systemic administration of a well-tolerated dose of 32-134D inhibits HIF and HIF-regulated gene expression and effectively treats retinal vascular disease in mice. (**A**) Representative fundus photos (above) and fluorescein angiographic images (below) of the retina of BL/6 mice on day 30 following 5 daily i.p. injections (day 0 to day 5) with 32-134D at 40 or 80 mg/kg compared with vehicle control. *n* = 3 animals (6 eyes) per group. (**B**) HIF-1α and HIF-2α protein accumulation at P13 and P14, respectively, 24 hours after a single i.p. injection with 32-134D (20 mg/kg) in OIR mice. (**C** and **D**) mRNA expression of HIF-regulated vasoactive mediators *Vegfa*, *Angptl4*, *Pdgfb*, and *Epo* (**C**) and endothelial cell genes *Angpt2*, *Ptprb*, *Serpine1*, and *Kdr* (**D**) in OIR mice treated with 5 consecutive (P12–P16) i.p. injections with 20 mg/kg 32-134D or vehicle control. (**E**) Left: representative images of retinal NV (outlined) at P17 after daily treatment with 20 mg/kg 32-134D or vehicle control (P12-16). Right: quantitation of avascular retina and retinal NV at P17. *n* = 3–8 animals per condition. Data are represented as mean ± SD. Statistical analyses were performed using 1-way ANOVA with Bonferroni’s multiple-comparison test (**C** and **D**) or 2-tailed Student’s *t* test (**E**). *P < 0.05; **P < 0.01; ***P < 0.001; ****P < 0.0001. Scale bars: 500μm.

Systemic administration of 32-134D by i.p. injections for 5 consecutive days at doses up to 80 mg/kg body weight has previously been reported to be well tolerated by adult mice, with no evidence of changes in appearance, behavior, body weight, hemoglobin, or hematocrit (44). Examination of the eyes from adult mice 1 month following 5 consecutive i.p. injections of 40 or 80 mg/kg 32-134D revealed a normal-appearing fundus without evidence of retinal vascular injury or leakage on fluorescein angiogram (Figure 10A). OIR mice treated with a single i.p. injection with as little as 20 mg/kg of 32-134D demonstrated a reduction in HIF-1α and HIF-2α protein accumulation (Figure 10B) at their respective peak expression at P13 and P14, respectively (14). Treatment of mice with 5 consecutive (P12–P16) i.p. injections with 20 mg/kg 32-134D resulted in a marked reduction in the mRNA expression of key HIF-regulated vasoactive mediators (Figure 10C), including those specifically expressed by vascular cells (Figure 10D). Similar results were observed in STZ mice that were hyperglycemic for 9 months and treated with 5 consecutive days of i.p. injections with 40 or 80 mg/kg 32-134D (Supplemental Figure 3). Accordingly, 5 consecutive days of i.p. injections with 20 mg/kg of 32-134D resulted in a marked reduction in retinal NV in OIR mice (Figure 10E).

### Intraocular administration of 32-134D does not affect retinal histology or function.

**Figure 11:**
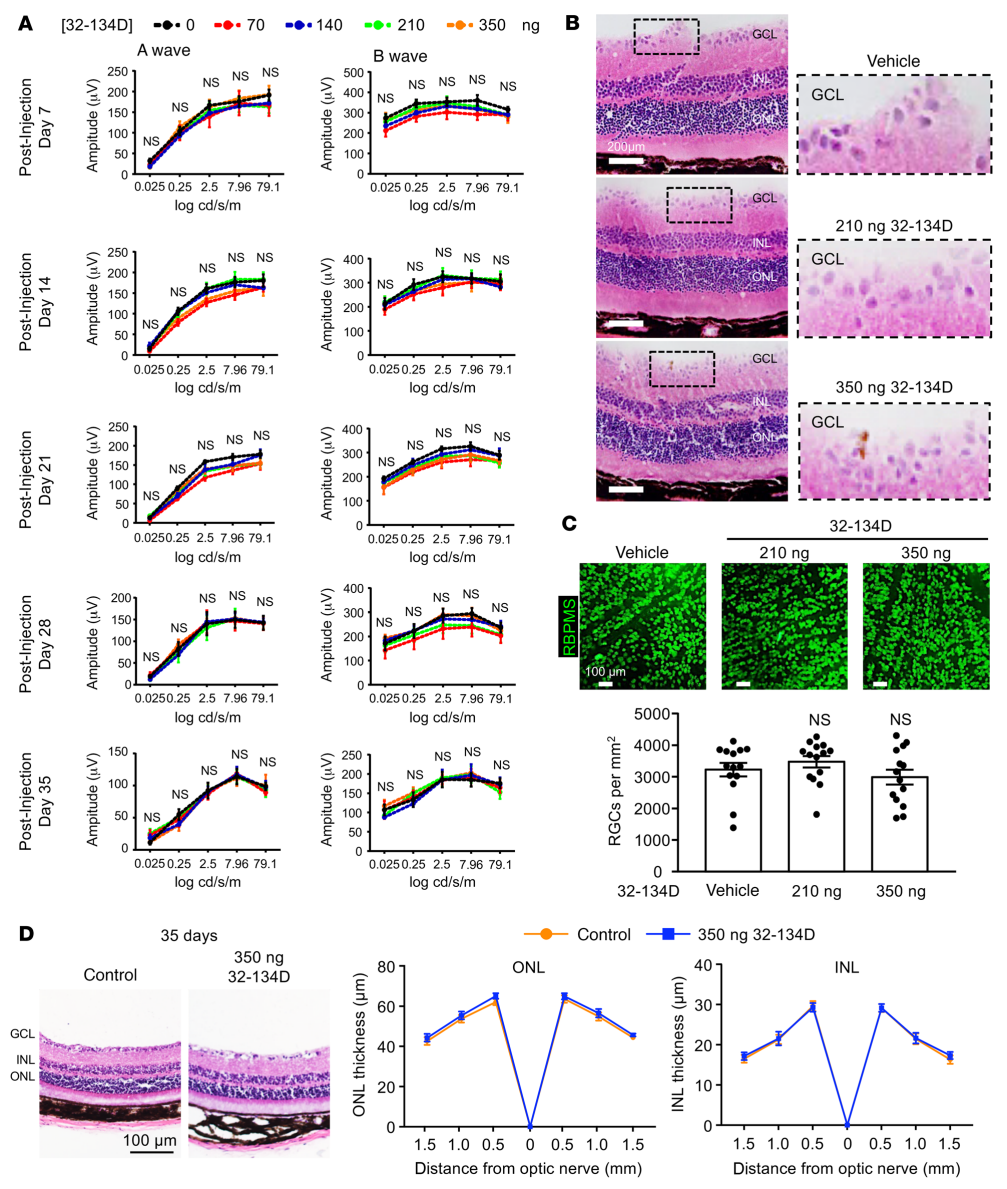
Intraocular administration 32-134D does not affect retinal function. (**A**) ERG of C57BL/6 mice 7 to 35 days following a single intraocular administration with vehicle or 32-134D at the stated doses. (**B**) Representative images of H&E staining of retinal sections 35 days following a single intraocular injection with 32-134D at the stated doses. (**C**) Top: representative images of RBPMS staining of RGCs in retinal flat mounts 35 days following a single intraocular injection with 32-134D at the stated doses. Bottom: quantitation of RGCs. (**D**) Left: representative images of H&E staining of retinal sections 35 days following a single intraocular injection with 32-134D at 350 ng. Right: quantitation of ONL and INL thickness. *n* = 5 animals for each group. Data are represented as mean ± SEM (**A**) or mean ± SD (**C** and **D**). Statistical analyses were performed by 2-way ANOVA (**A**) or 1-way ANOVA (**C**) with Bonferroni’s multiple-comparison test. Scale bars: 200 μm (**B**); 100 μm (**C** and **D**). Original magnification, ×20.

To reduce the risk of systemic side effects while optimizing the delivery of drugs to retinal tissue, therapies for ocular vascular disease are often administered by intravitreal injection. Therefore, we assessed whether 32-13D caused retinal toxicity following intraocular administration. To this end, we examined ERGs up to 5 weeks following a single intraocular injection of increasing doses (70, 140, 210, and 350 ng) of 32-134D, similarly to what was performed for acriflavine. Unlike with acriflavine, we did not observe any evidence of retina toxicity on ERGs at any of the doses of 32-134D tested (Figure 11A). Close examination of the eyes from mice treated with intraocular administration of 32-134D revealed normal histology, including a normal-appearing GCL (Figure 11B) 35 days following an intraocular injection with 210 or 350 ng of 32-134D. Quantitation of RGCs on retinal flat mounts demonstrated that the number of RGCs was unaffected by administration of 32-134D at the highest doses tested (Figure 11C). Similarly, careful measurements of the outer nuclear layer (ONL) or INL thickness 5 weeks after a single dose of 350 ng failed to demonstrate any changes compared with vehicle control (Figure 11D).

### Intraocular administration of 32-134D results in a sustained effective drug concentration in retinal tissue following a single injection.

**Figure 12:**
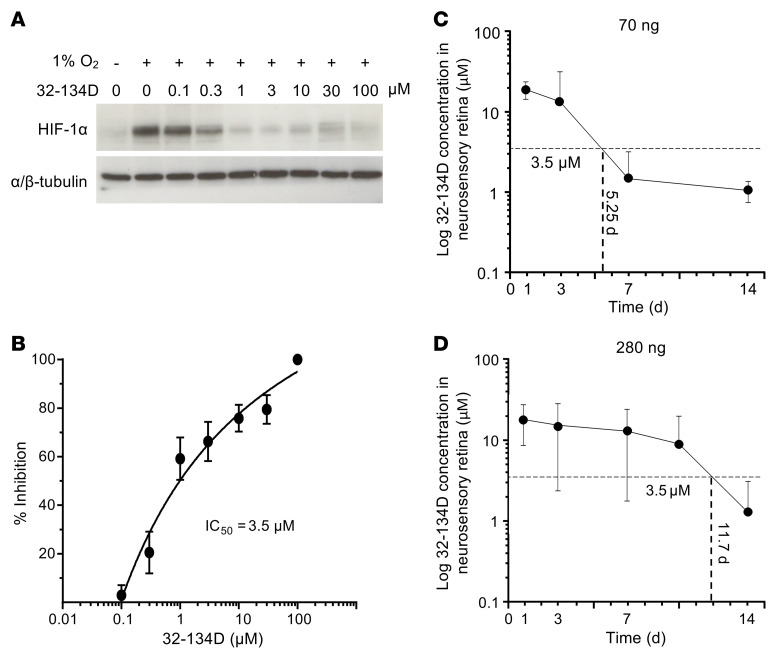
Calculations for effective dose of 32-134D following intraocular administration. (**A**) Accumulation of HIF-1α protein in MIO-M1 cells cultured in hypoxia and treated with 32-134D at indicated does. (**B**) Quantitation of immunoblots of HIF-1α protein accumulation in 32-134D–treated MIO-M1 cells cultured in hypoxia demonstrated an IC_50_ of 3.5 μM. (**C**) Concentration-time profiles of 32-134D in mice treated with a single intraocular injection with 32-134D (70 ng). The concentration of 32-134D in the neurosensory retina exceeded the in vitro IC_50_ of 3.5 μM for at least 5.25 days. (**D**) Concentration-time profiles of 32-134D in mice treated with a single intraocular injection with 32-134D (280 ng). Retina tissue was obtained over 14 days, with 32-134D concentrations determined by LC-MS/MS (**C** and **D**). The concentration of 32-134D in the neurosensory retina exceeded the in vitro IC_50_ of 3.5 μM for at least 11.7 days. *n* = 3 animals per condition. Data are represented as mean ± SD (**C** and **D**) and mean ± SEM (**B**).

To determine the appropriate dosing for intraocular administration of 32-134D in mice, we first examined its IC_50_ in MIO-M1 cells. Quantitation of immunoblots of HIF-1α protein accumulation in 32-134D–treated MIO-M1 cells cultured in hypoxia demonstrated an IC_50_ of 3.5 μM (Figure 12, A and B). To examine the pharmacokinetics of 32-134D in the neurosensory retina following intraocular administration, we employed liquid chromatography and tandem mass spectrometry (LC-MS/MS) to quantify the concentration of 32-134D in retinal tissue over 14 days following a single 70 ng intraocular injection of 32-134D (see Methods and Supplemental Methods, *Mouse pharmacokinetics*, for calculation of all parameters). The C_max_ achieved was 19.1 nmol/g at day 1 with apparent monoexponential decline. The total exposure (area under the curve [AUC_last_]) was 72.3 nmol × d/g. The concentration of 32-134D in the neurosensory retina exceeded the calculated in vitro IC_50_ of 3.5 μM for 5.25 days (Figure 12C). The t_1/2_ was not reportable due to the poor correlation coefficient (*r^2^* = 0.35).

To assess whether a higher dose of 32-134D could facilitate less frequent administration, pharmacokinetic analysis of 32-134D following intraocular administration was performed using a dose of 280 ng. Using LC-MS/MS to quantify the concentration of 32-134D in retina tissue over 14 days following a single intraocular injection of 280 ng, the C_max_ was 18 nmol/g in the neurosensory retina (Figure 12D), similar to the level achieved with 70 ng. However, the AUC_last_ was 147 nmol × d/g through 14 days, and the concentration of 32-134D in the neurosensory retina exceeded the in vitro IC_50_ of 3.5 μM for 11.7 days, both equating to double the effects of the 70 ng dose (Figure 12D). The t_1/2_ was 1.8 days at 280 ng.

### Gene expression changes following intraocular administration of 32-134D.

**Figure 13:**
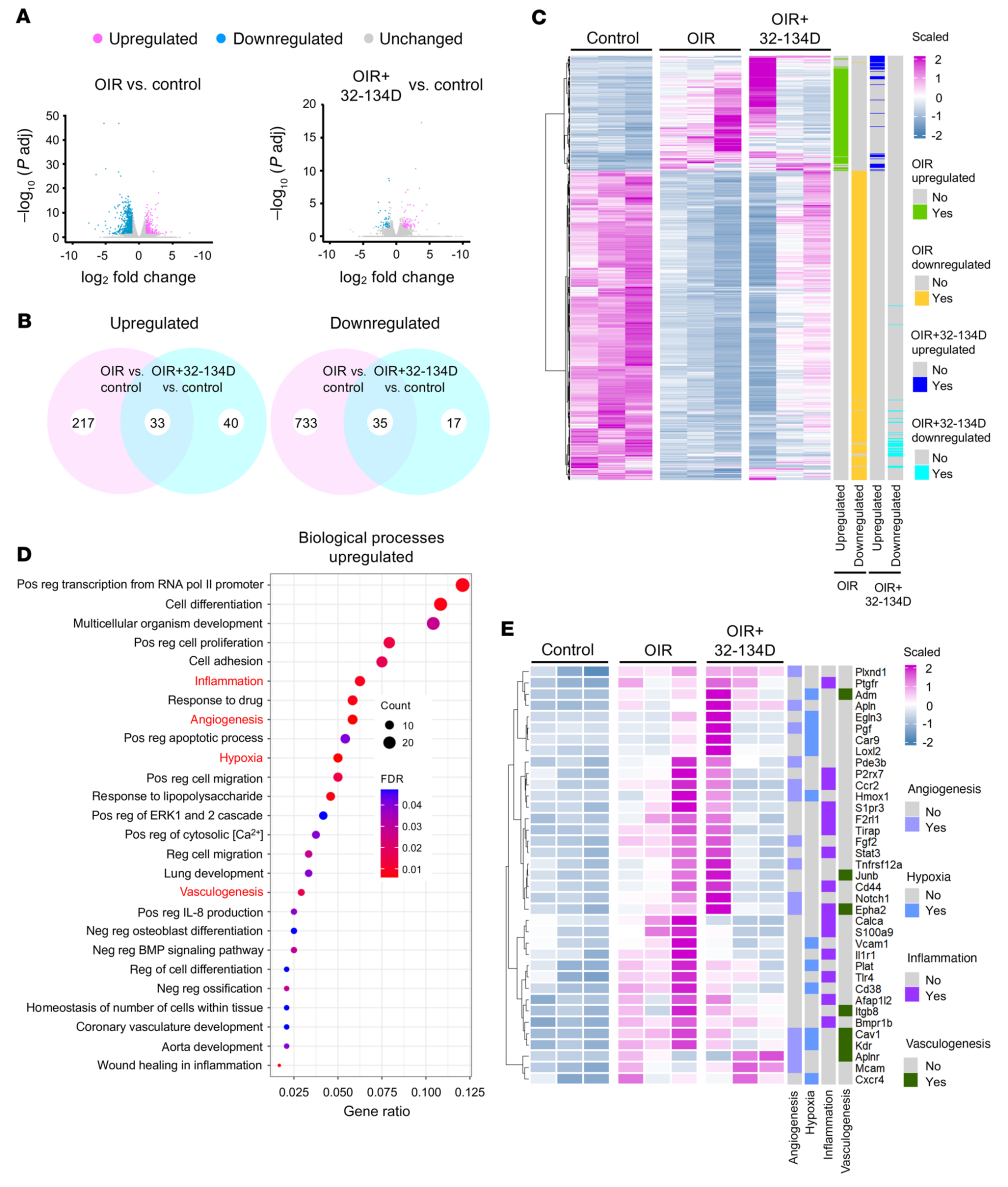
Gene expression changes following intraocular administration of 32-134D. Transcriptional analysis of retinal tissue of OIR mice at P17 following treatment with a single intraocular injection with DMSO (vehicle; OIR) or 32-134D (70 ng/μl; OIR+32-134D) at P12 compared with non-OIR (control) P17 mice. (**A**) Volcano plots illustrating DEGs in OIR versus non-OIR control mice (left) or OIR+32-134D versus OIR mice (right). (**B**) Venn diagrams showing overlap of 33 upregulated (left) and 35 downregulated (right) DEGs in OIR versus control (pink) and OIR+32-134D versus control (cyan), respectively. (**C**) Clustering analysis of identified DEGs among control, OIR, and OIR+32-134D. (**D**) GO analysis representing biological process enriched by 250 upregulated DEGs that were statistically significant (FDR < 0.05; red) or nonsignificant (FDR > 0.05; blue) between OIR and control. The size of the dots represents the count. The gene ratio describes the ratio of the count to the number of all DEGs. (**E**) Heatmap of top 37 upregulated DEGs in OIR and OIR+32-134D compared with control further enriched from 4 identified biological functions (highlighted in red in **D**). Bulk RNA-Seq analysis was performed from 3 independent isolations (*n* = 3 mice in each group). See Methods for details of statistical analyses performed. padj, adjusted P value; pos, positive; neg, negative; reg, regulation.

To assess its effects on gene expression, we performed RNA-Seq analysis at P17, 5 days following a single intraocular injection of 32-134D (70 ng/μL) in OIR mice at P12. Transcriptional analysis of neurosensory retinal tissue from P17 OIR mice demonstrated 250 upregulated and 768 downregulated differentially expressed genes (DEGs) compared with non-OIR control mice (Figure 13, A and B). Treatment with a single intraocular injection of 32-134D resulted in a marked reduction in both upregulated (73; 71% reduction) and downregulated (52; 93% reduction) DEGs compared with non-OIR (control) mice (Figure 13, B and C). Gene ontology (GO) analysis revealed over 2 dozen biological processes enriched by the 250 upregulated DEGs in OIR mice compared with control mice (Figure 13D). Among the biological processes that were significantly enriched (FDR < 0.05) were inflammation, angiogenesis, hypoxia, and vasculogenesis. These processes have previously been implicated in pathological angiogenesis in the OIR model and include many genes regulated by HIF-1 and HIF-2. A heatmap of the top 37 upregulated DEGs in OIR mice compared with control (non-OIR) mice demonstrated that treatment with 32-134D resulted in normalization of these 4 identified biological functions (Figure 13E). These data demonstrate that treatment with 32-134D helps normalize the expression of myriad HIF-regulated genes that influence numerous biological processes and collectively contribute to the development of retinal NV in ischemic retinal disease.

### Intraocular administration of 32-134D reduces retinal NV and vascular hyperpermeability in mouse models of diabetic eye disease.

**Figure 14:**
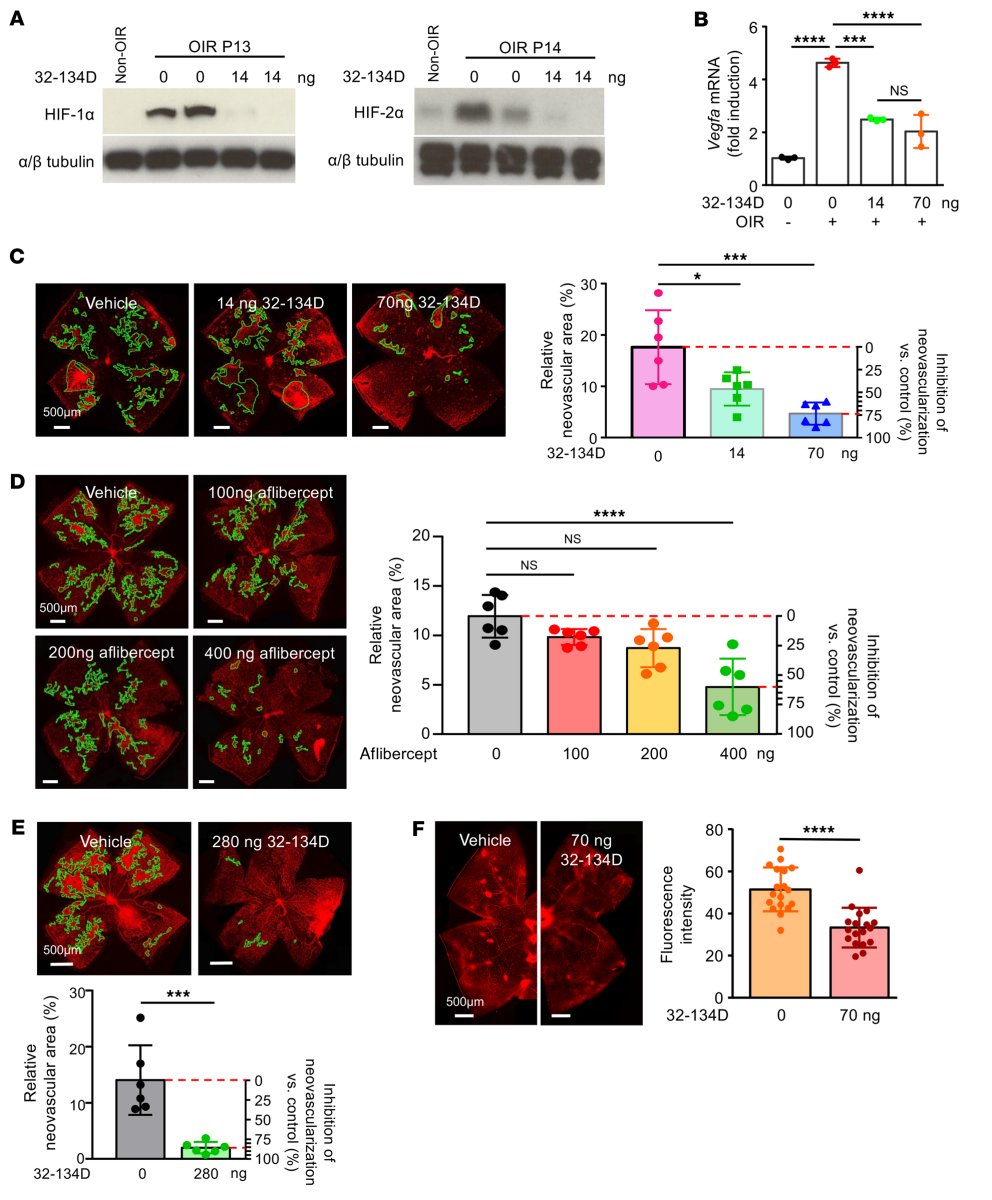
Intraocular administration of a well-tolerated dose of 32-134D reduces retinal NV and vascular hyperpermeability in mouse models of diabetic eye disease. (**A**) Western blot of HIF-1α and HIF-2α expression in OIR mice at P13 (left) and P14 (right), respectively, following a single intraocular injection with 32-134D (14 ng). (**B**) mRNA expression of *Vegf* in OIR mice treated with a single intraocular injection of 32-134D (14 or 70 ng) or vehicle control. (**C** and **D**) Left: representative images of retinal NV at P17 in OIR mice after a single intraocular injection of 32-134D (14 and 70 ng; **C**) or aflibercept (100, 200, or 400 ng; **D**) at P12. Right: quantitation (or reduction) of retinal NV compared with untreated control at P17. (**E**) Above: representative images of retinal NV at P17 in OIR mice after a single intraocular injection with 280 ng 32-134D at P12. Below: quantitation (or reduction) of retinal NV compared with untreated control at P17 in OIR mice after a single intraocular injection with 32-134D (280 ng). (**F**) Above: representative images of vascular hyperpermeability as demonstrated by leakage of intravascular Evans blue dye in STZ mice that were hyperglycemic for 6 months before treatment with 32-134D (or vehicle) 5 days prior to sacrifice. Below: quantitation of vascular hyperpermeability. *n* = 4–6 animals per condition. Data are represented as mean ± SD. Statistical analyses were performed using 1-way ANOVA with Bonferroni’s multiple-comparison test (**B**–**E**) or 2-tailed Student’s *t* test (**F**). *P < 0.05; **P < 0.01; ****P < 0.0001. Scale bar: 500 μm.

We next determined whether the modest but broad reduction in HIF-regulated gene expression following intraocular administration of 32-134D could affect retinal pathology. To this end, we performed a single intraocular injection with 32-134D at P12 and examined retinal NV at P17 in OIR mice. A single intraocular injection with 32-134D (14 ng) at P12 effectively reduced the accumulation of both HIF-1α and HIF-2α at their peak expression at P13 and P14, respectively (14) (Figure 14A). This, in turn, resulted in a moderate reduction in *Vegfa* mRNA expression (Figure 14B), but was sufficient to inhibit the development of retinal NV at P17 (by almost 75%) in OIR mice (Figure 14C), which was similar to what was observed with aflibercept (Figure 14D) at a dose equivalent to what is being used for newborns with retinopathy of prematurity (ROP) (47). This could be explained by the moderate reduction of a broad group of other key HIF-regulated angiogenic genes, including *Kdr*, *Angptl4*, *Epo*, and *Serpine1* (Supplemental Figure 4). Moreover, increasing the dose of 32-134D to 280 ng further inhibited the development of retinal NV (by approximately 85%) at a dose that was safe in treated mice (Figure 14E). Treating STZ mice that were hyperglycemic for 9 months with a single intraocular injection with 70 ng 32-134D also effectively inhibited vascular hyperpermeability (Figure 14F). Collectively, these data demonstrate that 32-134D is a safe and effective inhibitor of HIF activity, HIF-regulated gene expression, and, in turn, retinal vascular pathology (Supplemental Figure 5), with a wide therapeutic window following intraocular administration and that it may be more effective than current anti-VEGF therapies.

## Discussion

While anti-VEGF therapies have had a remarkable impact on the treatment of both PDR and DME, not all patients respond adequately despite monthly treatment, highlighting the importance of identifying new therapies for the prevention or treatment of diabetic eye disease. One approach for those patients who respond inadequately to current anti-VEGF agents is to design treatment modalities that inhibit VEGF more efficiently. However, these efforts may have unwanted consequences, given the proposed physiologic role of VEGF for the health of the neurosensory retina (48). An alternative approach has been the development of therapies targeting additional vasoactive mediators that could be used alone or in combination with current anti-VEGF drugs. While this approach has shown promise, the redundancy of vasoactive mediators in diabetic eye disease may limit the efficacy of therapies targeting a small subset of these factors. Indeed, we observed an increase in the levels of multiple angiogenic factors in patients with PDR or with NPDR and DME; importantly, these vasoactive mediators remained elevated despite treatment with anti-VEGF therapy. Determining which angiogenic mediators would be most effective as therapeutic targets for PDR or DME without compromising the health of the neurosensory retina remains an ongoing challenge.

Here, we explore a third strategy to more effectively treat patients with diabetic eye disease: to normalize the expression of multiple vasoactive factors back to their physiologic levels by inhibiting the transcriptional regulators that promote their pathologic increased expression (Supplemental Figure 5). Using animal models for ischemic retinal disease or for sustained hyperglycemia, we provide evidence that the transcription factors HIF-1 and HIF-2 regulate the expression of a broad spectrum of vasoactive mediators that have been implicated in diabetic eye disease. Pharmacologic inhibition of HIFs reduced the levels of these vasoactive factors to close to their baseline levels, preventing pathological angiogenesis without compromising the physiologic roles of these secreted proteins.

Interestingly, we observed that accumulation of HIF-1α in hyperglycemic mice occurs prior to — and independently of — the development of retinal ischemia (or hypoxia). Increased HIF expression in hyperglycemic mice was necessary to promote the expression of the HIF-regulated vasoactive mediators that promote vascular hyperpermeability. We have also recently reported that transient episodes of low glucose, as occurs in patients initially started on insulin treatment or those with high glycemic variability, result in the accumulation of HIF-1α independently of hypoxia (49). The accumulation of HIF-1α (and HIF-regulated vasoactive mediators) in hypoglycemia was markedly increased in the setting of even modest hypoxia. Collectively, these observations suggest that therapies directed against HIFs could be an effective approach to preventing the development or progression of early diabetic eye disease and demonstrate how targeting HIFs could effectively treat vascular permeability in patients with DME.

It has recently been reported that selective targeting of HIF-2 can inhibit retinal NV in the OIR mouse model of ischemic retinal disease (13, 14). It was further reported that expression of HIF-2α — but not HIF-1α — in vascular cells promotes the expression of PAI-1, an essential autocrine angiogenic factor in patients with PDR (13). It is therefore tempting to speculate that HIF-2–specific inhibition with belzutifan, recently approved by the FDA for the treatment of renal cell carcinoma in patients with von Hippel–Lindau disease, may be an effective approach for the treatment of ocular neovascular disease, including diabetic eye disease. However, using tissue from human eyes as well as hiPSC-derived retinal organoids, we reported that there is a redundancy of HIF-1α and HIF-2α expression in ischemic retina (14). While vascular cell expression of PAI-1 has been reported to be dependent on HIF-2 (13), Müller glial cell expression of ANGPTL4 was dependent on HIF-1 and expression of VEGF was dependent on both HIF-1 and HIF-2 (14). This suggests that targeting both HIF-1 and HIF-2 may be the most effective approach for blocking the expression of a broad spectrum of vasoactive mediators in patients with diabetic eye disease.

Several groups investigating the therapeutic potential of HIF-1 and HIF-2 inhibitors for the treatment of ocular vascular disease have focused on 3 pharmacologic inhibitors: digoxin, doxorubicin, and acriflavine, identified in drug screens performed 15 years ago (36, 40, 50). While an effective research tool, safely translating the cardiac glycoside digoxin to patients with ocular disease has proven to be challenging due in part to its complex pharmacokinetic profile and narrow therapeutic index (39). Indeed, intraocular administration of digoxin in rabbits at therapeutic doses has been reported to result in retinal toxicity (51, 52). Similar observations were made following intraocular administration of the chemotherapeutic agent doxorubicin (53, 54). Attention has therefore focused on acriflavine as the most promising of the 3 for the treatment of ocular vascular disease. Prior studies have demonstrated that acriflavine can effectively reduce retinal and choroidal NV in animal models using either intraocular or topical delivery (41). We demonstrate that free acriflavine can affect vascular hyperpermeability in diabetic mice for several months following a single intraocular administration. This prolonged biologic activity appears to be a consequence of its accumulation in the retina following intraocular administration, as has been previously reported (41, 55).

However, acriflavine is a fluorophore that intercalates into DNA and high–molecular weight RNA, raising concern for its safety for internal use in humans. Moreover, the DNA-damaging effects of acriflavine have been reported to be markedly increased in the presence of visible light exposure, a property directly linked to acriflavine’s DNA-intercalating properties (56), raising additional concerns about its use for the treatment of retinal disease. We report here that the accumulation of acriflavine in the retina in mice can cause retinal toxicity over time, with RGC loss at therapeutic doses. This appears to be due to off-target effects of acriflavine, as genetic reduction of HIF-1α or HIF-2α expression could not reproduce these results. Of note, the impact of the enhanced toxicity of acriflavine in the presence of visible light may be underestimated in the retinas of nocturnal animals, such as mice. While current efforts developing sustained-release formulations for acriflavine may help expand its therapeutic window (55, 57), these results raise concerns about its applications for the treatment of retinal diseases.

We therefore examined the safety and efficacy of an HIF inhibitor, 32-134D, which reproduces acriflavine’s inhibition of HIF-regulated genes, but is structurally distinct from acriflavine (44). Systemic administration of 32-134D has previously been reported to be very well tolerated by mice (44) at doses that we demonstrate prevent retinal NV without affecting normal retinal vasculature. Intraocular administration of a single dose of 32-134D inhibited HIF-1α and HIF-2α protein accumulation, thereby normalizing the expression of HIF-regulated vasoactive genes in mouse models of diabetic eye disease. These results were corroborated in hiPSC-derived 3D retinal organoids. Treatment of mice with a single intraocular injection of 32-134D effectively prevented the development of both retinal NV and vascular hyperpermeability at doses that did not affect the normal retinal histology or function. Interestingly, while C_max_ was not increased following an intraocular injection of 280 ng rather than 70 ng of 32-134D, there was a depot-like effect observed that more than doubled the time (from 5.25 to 11.75 days) during which 32-134D levels were above the IC_50_. This may facilitate an extended interval between injections for patients receiving intraocular injections with 32-134D in the clinic. Further studies are required to determine the optimal dose and frequency of treatment needed to achieve steady-state levels of 32-134D that are above its IC_50_.

By targeting both HIF-1 and HIF-2, we demonstrate that 32-134D effectively inhibited genes implicated in over 2 dozen biological processes, including inflammation, angiogenesis, hypoxia, and vasculogenesis, which have all previously been implicated in pathological angiogenesis in ischemic retinal disease. Among the angiogenic signaling cascades inhibited by 32-134D are the VEGF/KDR axis (currently targeted by the FDA-approved therapies for DME and PDR, ranibizumab, aflibercept, and brolucizumab, as well as the oncology drug bevacizumab, used off-label for the treatment of diabetic eye disease) as well as the ANGPT2/TIE2/VE-PTP cascade (currently targeted by faricimab, a bispecific antibody targeting both VEGF and ANGPT2 [refs. 58, 59], and AKB-9778, a subcutaneous VE-PTP inhibitor [refs. 60, 61]). By normalizing the expression of both the vasoactive mediator (e.g., VEGF and ANGPT2) and its downstream effectors (e.g., KDR and VE-PTP), 32-134D may effectively inhibit these pathways even though the levels of expression of these proteins do not fall significantly below their normal physiologic levels.

Treatment with 32-134D also inhibited the expression of many other angiogenic mediators that have been previously implicated in ischemic retinal eye disease (4) and may further contribute to the reduced response of some patients to current anti-VEGF therapies (13, 18, 62). This highlights a central advantage of therapies targeting HIFs over those therapies that target a specific vasoactive mediator regulated by HIFs: by modestly reducing the expression of a broad spectrum of angiogenic and hyperpermeability factors back to their physiological levels, 32-134D may effectively treat ischemic retinal eye disease while reducing the risk for adverse outcomes that can result from the chronic suppression of 1 or 2 vasoactive mediators to subphysiological levels. HIF inhibition may also be effective for retinal vascular diseases in which HIFs and/or retinal ischemia plays a central role, but for which anti-VEGF therapies have proven inadequate, including von Hippel–Lindau disease (63, 64), Coats disease (65, 66), Norrie’s disease (67, 68), and familial exudative vitreoretinopathy (FEVR) (69). Collectively, these results provide the foundation for the early clinical assessment for the use of 32-134D for the treatment of ocular vascular disease.

## Methods

### Cell culture and reagents.

MIO-M1, HUVECs, and ARPE19 cells were cultured in DMEM with 10% (vol/vol) FBS (Quality Biological) and 1% penicillin/streptomycin (Cellgro). A hypoxic chamber (Coy Laboratory Product Inc.; Two Glove Model) with 5% CO_2_, 94% N_2_, and 1% O_2_ gas concentration at 37°C was used to culture hypoxic cells. The control cells were cultured in an incubator maintained at 37°C, with 21% O_2_ and 5% CO_2_. Digoxin was purchased from Tocris (4583/50). Acriflavine was obtained from MilliporeSigma (8048-52-0). DMSO (472301-500ML) and Evans blue (E2129) were purchased from Sigma-Aldrich, and 4-(6-bromo-1H-indol-3-yl)-2-(7-bromo-1H-indol-3-yl)thiazole, designated 32-134D, was synthesized as previously described (44). Aflibercept was obtained from the Johns Hopkins University Pharmacy.

### Retinal organoids.

An hiPSC line derived from CD34^+^ cord blood was used in this study (A18945, Thermo Fisher Scientific) (70). Undifferentiated hiPSCs and derived retinal organoids were routinely tested for mycoplasma contamination by PCR. Cell culture, retinal differentiation, and organoid formation were conducted as previously described (71). Retinal organoids at D120 were used for experiments.

### Cell-based studies.

Please see details for all cell-based studies in Supplemental Methods.

### Animal studies.

Please see details for all animal studies in Supplemental Methods.

### Human tissue studies.

Please see details for all human tissue studies in Supplemental Methods.

Descriptions of antibodies (Supplemental Table 1) and primer sequences (Supplemental Table 2) used in this study can be found in Supplemental Methods. See supplemental material for full, uncut gels.

### Data availability.

All data will be made available upon request. RNA-Seq data have been deposited at NCBI’s Gene Expression Omnibus (GEO GSE229730). Values for all data points found in graphs can be found in the supporting data values file.

### Statistics.

Statistical analyses for the angiogenesis arrays and bulk RNA-Seq are described above. For all other cases, results are shown as mean ± SD or mean ± SEM from at least 3 independent experiments. Statistical analysis was performed with Microsoft Excel, version 16.0.16327.20248, or Prism, version 8.0, software (GraphPad). Statistical differences between 2 or multiple heterogenous groups were determined by unpaired Student’s *t* test or 1-way or 2-way ANOVA. Analysis of data was performed using Excel.

### Study approval.

All animal experiments were performed in accordance with the Animal Care and Use Program at Johns Hopkins University and were approved by the Institutional Animal Care and Use Committee (IACUC). All studies involving patients or patient tissue were approved by the Institutional Review Board of Johns Hopkins University School of Medicine. All participants provided written informed consent prior to enrolment in the study.

## Author contributions

AS is the primary contributor to research design. JZ, DS, AD, JS, BPA, KJ, MD, MFB, MWH, CG, SS, YH, and NMA were responsible for research execution and were contributors to data acquisition. AS, JZ, DS, AD, BPA, KJ, MD, MFB, MWH, CG, NMA, MAR, MVCS, JQ, GLS, and SM were the primary contributors to data analysis and interpretation. Manuscript preparation was by AS, with revisions provided by JZ, DS, MAR, MVCS, GLS, and SM.

## Supplementary Material

Supplemental data

Supporting data values

## Version 1 05/25/2023

In-Press Preview

## Version 2 07/03/2023

Electronic publication
