# Supplementary material for: Targeting hypoxia-inducible factors with 32-134D safely and effectively treats diabetic eye disease in mice
Source: J Clin Invest. 2023 Jul 3;133(13):e163290. doi: 10.1172/JCI163290 (PMC10313368; doi:10.1172/JCI163290)

Supplemental Table 1. Antibodies used in this study.

Name	Company	Species	Catalog Number	dilution	Applications
HypoxyProbe	HPI	rabbit	PAb2627AP	1:50	IF
Isolectin GS IB4	Thermo Fisher		I21413	1:200	IF
HIF-1α	Gene Tex	rabbit	GTX127309	1:1000	WB
HIF-2α	Gene Tex	mouse	GTX632015	1:1000	WB
VEGF	Santa Cruz	rabbit	sc-152	1:1000	IHC
ANGPTL4	Abcam	rabbit	ab115798	1:400	IHC
α/β tubulin	Cell signaling	rabbit	2148s	1:5000	WB
HIF-1α	Abcam	rabbit	ab2185	1:500	IHC
RBPMS	Abcam	rabbit	ab152101	1:200	IF
CRALBP	Abcam	mouse	ab15051	1:500	IF
REC	MilliporeSigma	rabbit	AB5585	1:500	IF
PAX-6	DSHB	mouse	AB_528427	1:50	IF
secondary antibodies	Invitrogen			1:1000	IF
secondary antibodies	Dako			1:100	IHC

Abbreviations: IF, immunofluorescence; WB, Western Blot; IHC, immunohistochemistry

Supplemental Table 2. Primer sequences for Real-Time (RT)-PCR

	Gene		Sequence (5'to 3')
Mouse			
	Angptl4	Forward	TTGGTACCTGTAGCCATTCC
		Reverse	GAGGCTAAGAGGCTGCTGTA
	Vegfa	Forward	TCCTCCTATCTCCACC
		Reverse	GACCCAGCCAGCCATA
	Ppia	Forward	AGCATACAGGTCCTGGCATC
		Reverse	TTCACCTTCCCAAAGACCAC
	Serpine1	Forward	TGATGGCTCAGAGCAACAAG
		Reverse	GCCAGGGTTGCACTAAACAT
	Angpt2	Forward	TCCAAGAGCTCGGTTGCTAT
		Reverse	AGTTGGGGAAGGTCAGTGTG
	Ptprb	Forward	TCAAGGCAGGACAGTACCC
		Reverse	TGTATTTCTCCCATTCGCCTAGA
	Pdgfb	Forward	TGCTGCACAGAGACTCCGTA
		Reverse	GATGAGCTTTCCAACTCGACTC
	Kdr	Forward	GCAGAAGATACTGTCACCACC
		Reverse	TTTGGCAAATACAACCCTTCAGA
	Epo	Forward	ACTCTCCTTGCTACTGATTCCT
		Reverse	ATCGTGACATTTTCTGCCTCC
Human			
	ANGPTL4	Forward	GGACACGGCCTATAGCCTG
		Reverse	CTCTTGGCGCAGTTCTTGTC
	VEGFA	Forward	GGGCAGAATCATCACGAAGT
		Reverse	TGGTGATGTTGGACTCCTCA
	PPIA	Forward	TTCATCTGCACTGCCAAGAC
		Reverse	TCGAGTTGTCCACAGTCAGC
	SERPINE1	Forward	ACAACAGGAGAAACCCA
		Reverse	CACGTCATGGGTGGTTTCTTG
	ANGPT2	Forward	GCAAGTGCTGGAGAACATCA
		Reverse	GTTAACTTC CGCGTTTGCTC
	PTPRB	Forward	ACAACACCACATACGGATGTAAC
		Reverse	CCTAGCAGGAGGTAAAGGATCT

Supplemental Materials

Cell-based studies.

Immunoblot assays. Cells, neurosensory retina, and D120 retinal organoid lysates were subjected to 4–15% gradient SDS/PAGE (Invitrogen). Immunoblot (Western blot or WB) assays were performed as previously described (1).

Reverse transcription and quantitative real-time PCR. Total RNA was isolated from cultured cells or retinas with PureLink™ RNA Mini Kit (Invitrogen #12183025), and cDNA was prepared with MuLV Reverse Transcriptase (Applied Biosystems). Quantitative real-time PCR was performed with Power SYBR Green PCR Master Mix (Applied Biosystems) and MyiQ Real-Time PCR Detection System (Bio-Rad). Normalization was done using cyclophilin A for mouse tissue and human cell lines. Primers are listed in Supplemental Table 2.

Angiogenesis array. Total RNA was isolated from MIO-MI cells (8 hours) and HUVECs (16 hours) cultured in normoxia (control), hypoxia without 32-134D (hypoxia), and in hypoxia with 32-134D (hypoxia + 32-134D) with PureLinkTM RNA Mini Kit (Invitrogen, Cat#12183025) and cDNA was prepared with QuantiTect® Reverse Transcription Kit (Qiagen, Cat# 205311). RT-qPCR assay was performed with pre-configured TaqMan Array 96-Well FAST Plate for human angiogenic gene expression (Applied Biosystems, Inc) following the manufacture's protocol. Each plate contains pre-defined genes including *18S*, *GAPDH* and *HPRT1* endogenous controls. TaqMan Array plates were prepared by loading on each well 5 µl of cDNA sample (30 ng) with nuclease-free water and 5 µl of TaqMan™ Fast Advanced Master Mix (Applied Biosystem, Cat#

4444554). The reaction was performed using the QuantStudio™ 3 Flex Real-Time PCR System as follows: 2 minutes at 50°C (stage 1); 10 minutes at 95°C (stage 2); 15 seconds at 95°C and 1 minutes at 60°C for 40 cycles (stage 3). The data were analyzed by using *HPRT1* as endogenous controls.

The clustering heatmaps were produced with "pheatmap" library in R with scale = row and clustering method = complete. For illustrating the response of 32-134D treatment in MIO-MI and HUVEC cells, Euclidean distance was used to quantify the difference in qPCR screening. The Euclidean distance was calculated using "dist" function from R's default stat library that measures the distance between 2 vectors (i.e., total qPCR expression values in control and treatment). In MIO-MI cells, the Euclidean distances were 73 between control and hypoxia, and 31 between control and hypoxia + 32-134D. In HUVEC cells, the Euclidean distances were 208 between control and hypoxia, and 35 between control and hypoxia + 32-134D.

Animal studies. Eight-week-old pathogen-free female C57BL/6 mice were obtained from The Jackson Laboratory (JAX). Timed pregnant C57BL/6 mice were obtained from Charles River Laboratories. Mice heterozygous for HIF-1α have been previously described (2). Mice heterozygous for HIF-2α (strain number: 003266; common name: EPAS1 KO) were purchased from JAX.

OIR mouse model. OIR experiments were performed as previously described (1, 3). Briefly, C57BL/6 mice were placed in 75% O_2 on P7. On P12, the mice were returned to room air and were either administered digoxin and 32-134D (or vehicle) by IP injections or 32-134D or acriflavine (or vehicle) by intravitreal injection. Mice with body weight less than 6 g at P17 were excluded

from analysis. Data were collected from both males and females and the results were combined since there was no apparent difference between sexes. Representative images for selected time points from a minimum of three independent experiments are shown. Data from 3-8 pups were obtained at each time point.

STZ-induced diabetes mouse model. 8-to-10 week-old male mice received an IP injection of STZ at 40 mg/kg of body weight in 0.1M of citrate buffer (pH 4.5) for 5 consecutive days. Normal chow and 10% sucrose water were provided during this period. 10% sucrose was discontinued and replaced with regular water on experimental day 6. Blood glucose was measured on experimental day 28 after 16 hours fasting and mice with blood glucose level > 250 mg/dL were considered diabetic.

Intraperitoneal (IP) injections. Digoxin (2 mg/kg) and 32-134D (20 to 80 mg/kg) were injected intraperitoneally using an insulin syringe in OIR pups. Digoxin and 32-134D were injected into the peritoneal cavity of neonatal C57BL/6 mice and STZ mice at indicated times, and the retinal vasculature and lysates for WB and qPCR were assessed at P17.

Intraocular injections. Intravitreal injections were performed with a PLI-100A Pico-liter Microinjector (Warner Instruments, Harvard Bioscience) using pulled-glass micropipettes. Each micropipette was calibrated to deliver a 1 µl volume on depression of a foot switch. The mice were anesthetized with a ketamine (100 mg/kg) and xylazine (5mg/kg) mixture, and under a dissecting microscope, the sharpened tip of the micropipette was passed through the sclera just posterior to the limbus into the vitreous cavity and the foot switch was depressed, which caused fluid to

penetrate into the vitreous space. 32-134D was injected into the vitreous cavity in OIR and STZ mice for vascular permeability test, retinal vasculature, and lysates for WB and qPCR analysis.

Immunofluorescence assays. Details for antibodies are provided in Supplemental Table 1. Immunofluorescence assays in retinal organoids and mouse retinal tissues were performed as previously described (4). Briefly, mice were sacrificed using CO_2 asphyxiation, and the eyes were enucleated and fixed with a solution of 4% paraformaldehyde in PBS (Thermo Scientific™) for two hours at RT, followed by washing with PBS for 10 minutes in a shaker. Retinas were isolated and incubated in 0.5% BSA solution overnight at 4ºC. Retinas were then washed with PBS 3 times for 10 minutes in the shaker and stained with isolectin B4 (Invitrogen; 1:200 dilution in PBS) overnight at 4ºC. After washing 3 times for 10 minutes each in the shaker, the retinas were mounted. Images were captured with Zeiss fluorescent microscope. Eight fields per eye (2 fields per petal) including area of neovascularization were selected and fluorescence intensity was measured by ImageJ software.

Vascular permeability by Evans blue tracer method. Mice were anesthetized with a mixture of ketamine (50 mg/kg) and xylazine (5mg/kg) and subjected to Evans blue (Sigma) (50mg/kg) perfusion by penile vein injection. After 10 minutes, animals were sacrificed by CO_2 asphyxiation. The eyes were enucleated and immediately immersed in 4% (wt/vol) paraformaldehyde for 2 hours at room temperature. Retinas were isolated and wholemounts prepared on clean glass slides and mounted vitreous side up under coverslips with antifade medium (Vectashield; Vector Laboratories, Burlingame, CA). Images were captured by Zeiss fluorescent microscope or confocal microscopy and fluorescence was quantified by the image J software (National Institutes

of Health, Bethesda, MD). Blinded quantification of the number of leaking vessels was performed by manual counting of major leakage from capillaries from retinas of each group. Non-capillary artifactual Evans blue or FITC-dextran leakage due to flat mount processing as well as minor leakages were not included in the quantitation. Quantification of fluorescence intensity was performed by the ImageJ software by selecting leakages from each petal of retina when leakage was present; in the absence of leakage, random images were taken of each petal of retina. Random fields were chosen from retinas where leakages were absent to determine baseline fluorescence that arise from capillaries. High magnifications (20X) images were used for quantification. Images of whole flat mount retinas included in figures were taken using low magnification (5X).

Hypoxia in mouse tissue. Hypoxia detection in mouse tissue sections was performed using Hypoxyprobe™ Kit (HPI Catalog#: PAb2627AP). Hypoxia assays were performed according to the manufacturer's recommendations. Pimonidazole HCl (60 mg/kg) was injected intraperitoneally in experimental mice and eyes were collected after 90 minutes injection for immunofluorescence assays. Tissue specimens were collected and directly frozen in liquid nitrogen until cryosectioning into 4-μm sections. Consecutive sections were cut at the largest circumference of the tissue. The sections were then stored at -80°C until they were stained. After thawing, the sections were fixed in cold acetone (4°C) for 10 minutes. The sections were then rinsed and incubated overnight at 4°C with mouse monoclonal anti-pimonidazole antibody (clone 4.3.11.3; MAb1) diluted in PBS containing 0.1% bovine serum albumin and 0.1% Tween 20. The sections were then incubated for 90 minutes with Cy-3-conjugated goat anti-mouse antibody (Jackson Immuno Research Laboratories) diluted 1:150. Between each step of the staining

protocol, the sections were rinsed three times with PBS for 2 minutes. Images were captured at high magnification (20X) with Zeiss fluorescent microscope.

Flash scotopic ERGs. All procedures were performed under dim red light. Mice were dark adapted overnight, anesthetized with ketamine (50 mg/kg) and xylazine (5mg/kg) and pupils were dilated with a topical drop of tropicamide (1%). Scotopic ERG responses were measured using the Celeris ERG stimulator (Diagnosys, Lowell, MA) at flash intensities of 0.025, 0.25, 2.5, 7.96, and 79.06 cd/s/m^2. ERGs were measured simultaneously from both eyes with a ground electrode placed into the forehead between the eyes and a reference electrode into the hip.

Fluorescein angiography in mice. To evaluate the effect of 32-134D on the retinal vasculature and vascular permeability, experimental animals were treated with 5 consecutive daily IP injections with 32-134D at 40 or 80 mg/kg. On day 30, mice were anesthetized using intramuscular injection with ketamine (50 mg/kg) and xylazine (5mg/kg). Pupils were then dilated using 1% tropicamide eye drops and mice were placed on the imaging platform of the Phoenix Micron III retinal imaging microscope (Phoenix Research Laboratories, Pleasanton, CA). Goniovisc 2.5% (hypromellose; Sigma Pharmaceuticals, LLC, Monticello, IA) was applied liberally to keep the eye moist during imaging studies. Fundus photos were taken before administering 10 to 20 µl of 10% fluorescein sodium (Apollo Ophthalmics, Newport Beach, CA) IP injection. Rapid acquisition of fluorescein angiographic images was then performed for 5 minutes. Fluorescein leakage manifests as indistinct vascular borders progressing to diffusely hazy fluorescence. Fluorescein leakage was compared between different groups by quantifying the fluorescence intensities collected after 1-, 2-, and 3-minutes following fluorescein injection using the ImageJ software.

Mouse pharmacokinetics. C57BL/6 mice were administered 32-134D at 70 ng or 280 ng as a single intraocular injection. Mice (at least 3 mice per time point) were euthanized at 1, 3, 7, or 14 days after injection. For the 280-ng dose, a 10-day time point was added. 32-134D was quantified in retina by LC-MS/MS as previously described (5) with the following modifications. Retina tissue samples were homogenized in 200 µL of 1× PBS (pH 7.4) before extraction. The standard curve and quality control samples were prepared in 1× PBS as a surrogate matrix. Retina tissue samples were then quantitated in µM as: nominal concentration (µM) × initial dilution ([tissue weight (mg) + volume of solvent (µl)]/ tissue weight (mg)). For all samples <10 nM, the value was reported as below the limits of detection. If at least on samples was >10 nM and one was <10 nM, then one half that value (5 nM) was imputed to calculate the concentration for that specimen and utilized in the average and standard deviation calculations.

Pharmacokinetic parameters were calculated from mean concentration-time data using noncompartmental methods in Phoenix WinNonlin version 8.3 (Certara, Princeton, NJ, USA). The C_{max} and time to C_{max} (T_{max}) were the observed values. The AUC_{last} was calculated using the log-linear trapezoidal method to the last quantifiable time point. The λ_z was determined from at least 3 points on the slope of the terminal phase of the concentration-time profile using a $1/y^2$ weighting factor. The $T_{1/2}$ was determined by dividing 0.693 by λ_z. $T_{1/2}$ was not reported if the correlation coefficient (r^2) for λ_z was less than 0.9. The total time above the IC_{50} (3.51 nmol/g) was calculated.

Bioanalytical method for mouse pharmacokinetics. Retina tissue samples were homogenized in 200 µL of 1× PBS (pH 7.4) before extraction. The standard curve and quality control samples were prepared in 1× PBS as a surrogate matrix for all matrices. Tissue homogenate or PBS (25 µL) was

added to a borosilicate glass test tube and mixed with 150 µL of acetonitrile containing the internal standard (1 ng/mL of EXP-3179). For blank samples, 150 µL of acetonitrile was added without internal standard. Samples were vortex-mixed and centrifuged (1200 ×g for 5 minutes at ambient temperature) and transferred to an autosampler vial. Then 2 µL was injected onto the liquid chromatography system using a temperature-controlled autosampling device operating at approximately 10°C. Chromatographic analysis was performed using a Waters Acquity TM Ultra Performance LC. Separation of the analyte from potentially interfering material was achieved at ambient temperature using Halo C18 column (50 x 2.1 mm i.d.) with a 2.7-µm particle size. The mobile phase used for the chromatographic separation was composed of 0.1% (v/v) formic acid in water (mobile phase A) and 0.1% (v/v) formic acid in acetonitrile (mobile phase B) with a flow rate of 0.4 mL/minute. The initial mobile phase composition was 60% mobile phase A and 40% mobile phase B. From 0.5 to 2.0 minutes, mobile phase B was increased linearly from 40% to 100% and maintained until 3.0 minutes. From 3.0 to 3.1 min, the gradient decreased to 40% mobile phase B and the conditions were maintained until 5 minutes to re-equilibrate the column for the next injection. The column effluent was monitored using an AB Sciex Triple Quadrupole 5500 mass spectrometer. The instrument was equipped with an electrospray interface, operated in a positive mode and controlled by the Analyst v1.7 software. The settings were as follows: curtain gas 20 psi, medium collision gas, ion spray voltage 5500 V, probe temperature 450°C, ion source gas one 30 psi, ion source gas two 40 psi, and entrance potential 10. The collision cell exit potentials were 14.0 and 6.0 for 32-134D and the internal standard, respectively. The declustering potential was 141 and 80 for 32-134D and the internal standard, respectively. The collision energies were 43 and 25 for 32-134D and internal standard, respectively. MRM m/z transitions were the following: 474.6 → 393.8 and 421.0 → 207.1 for 32-134D and the internal standard,

respectively. Dwell time was 150 milliseconds. The calibration curve for 32-134D was constructed from the peak area ratio of the analyte to the peak area of its internal standard (EXP-3179) using the least-squares quadratic regression analysis with 1/x2 weight over the range of 10-2,110 nM with dilutions of up to 1:10 (v/v).

Bulk RNAseq. P17 mouse pups from non-OIR (control), OIR injected with DMSO (vehicle) at P12 (OIR) and OIR injected with 70 ng/µl of 32-134D at P12 (OIR + 32-134D) were euthanized, enucleated, and their retinas separated from the RPE/choroid. Total RNA was isolated using miRNeasy mini kit (QIAGEN). RNA was quantified using Nanodrop (ND-1000 spectrophotometer; Thermo Scientific). The RNA samples were sequenced by Novogene Corporation Inc.

Raw data (fastq files), data quality control, and genome alignment were generated by Novogene company. DESeq2 (6) was used for differentially expressed genes (DEG) analysis. Genes with adjusted $P < 0.05$ and log2 fold change > 1 were identified as DEGs. Normalized counts were used to produce clustering heatmaps and "complete" was the default method in the clustering analysis. Gene Ontology (GO) analysis (biological process) was done using DAVID Bioinformatics Resources (7, 8). FDR < 0.05 was used as the threshold for enriched biological process.

Human tissue studies.

Patient selection and samples. Undiluted aqueous or vitreous aspirates were collected from consenting patients at the Wilmer Eye Institute undergoing vitrectomy or cataract surgery as previously described (9, 10). Consent was written, voluntary, and without stipend. Inclusion criteria included any patient with diabetes undergoing vitrectomy with active proliferative diabetic

eye disease (PDR samples) or undergoing intravitreal injections for DME (DME samples) for vitreous and aqueous samples, respectively. Non-diabetic patients undergoing vitrectomy surgery for vitreous floaters or epiretinal membrane (aqueous and vitreous samples) or cataract surgery (aqueous samples) were included as controls. Exclusion criteria included other ischemic retinal disease, uveitis, retinal detachment within 1 year of sample collection, or neovascularization or macular edema from another cause. Samples were immediately centrifuged at 16,000 x g for 5 minutes at 4°C, and then stored at -80°C prior to analysis. Imaging studies (fluorescein angiogram and spectral domain optical coherence tomography) from representative study patients were included to illustrate typical microvascular changes observed in patients with PDR or DME.

ELISAs. Levels of secreted VEGF, ANGPTL4, ANGPT2, EPO, and MMP2 were measured in vitreous and aqueous from patients using human ELISA kits (R&D System) according to the manufacturer's recommendations. Vitreous was diluted 1:10 for the ELISAs. Aqueous was undiluted. ELISAs are representative of at least three independent experiments.

Immunohistochemistry. Details for antibodies are provided in Supplemental Table 1. Immunohistochemical detection of human paraffin-embedded tissue sections using premixed biotinylated anti-rabbit, anti-mouse, and anti-goat immunoglobulins in phosphate buffered saline (PBS) from an ABC system (Dako, Santa Clara, CA) was performed according to the manufacturer's protocols as previously described (11, 12). All immunohistochemical reagents, including antibodies, were identical for all specimens. Images were captured by scanning slides using the Aperio ScanScope program on Aperio Scanscope XT1 System (Leica Biosystems, Wetzlar, Germany).

Supplementary References

1. Xin X, Rodrigues M, Umapathi M, Kashiwabuchi F, Ma T, Babapoor-Farrokhran S, et al. Hypoxic retinal Muller cells promote vascular permeability by HIF-1-dependent up-regulation of angiopoietin-like 4. *Proceedings of the National Academy of Sciences of the United States of America.* 2013;110(36):E3425-34.
2. Yu AY, Shimoda LA, Iyer NV, Huso DL, Sun X, McWilliams R, et al. Impaired physiological responses to chronic hypoxia in mice partially deficient for hypoxia-inducible factor 1alpha. *J Clin Invest.* 1999;103(5):691-6.
3. Rodrigues M, Xin X, Jee K, Babapoor-Farrokhran S, Kashiwabuchi F, Ma T, et al. VEGF secreted by hypoxic Muller cells induces MMP-2 expression and activity in endothelial cells to promote retinal neovascularization in proliferative diabetic retinopathy. *Diabetes.* 2013;62(11):3863-73.
4. Zhang J, Qin Y, Martinez M, Flores-Bellver M, Rodrigues M, Dinabandhu A, et al. HIF-1alpha and HIF-2alpha redundantly promote retinal neovascularization in patients with ischemic retinal disease. *J Clin Invest.* 2021;131(12).
5. Salman S, Meyers DJ, Wicks EE, Lee SN, Datan E, Thomas AM, et al. HIF inhibitor 32-134D eradicates murine hepatocellular carcinoma in combination with anti-PD1 therapy. *J Clin Invest.* 2022;132(9).
6. Love MI, Huber W, and Anders S. Moderated estimation of fold change and dispersion for RNA-seq data with DESeq2. *Genome Biol.* 2014;15(12):550.
7. Huang da W, Sherman BT, and Lempicki RA. Systematic and integrative analysis of large gene lists using DAVID bioinformatics resources. *Nat Protoc.* 2009;4(1):44-57.

8. Huang da W, Sherman BT, Zheng X, Yang J, Imamichi T, Stephens R, et al. Extracting biological meaning from large gene lists with DAVID. *Curr Protoc Bioinformatics.* 2009;Chapter 13:Unit 13 1.

9. Applewhite BP, Babapoor-Farrokhran S, Poon D, Hassan SJ, Wellmann E, Ying HS, et al. Lack of Evidence for Vasoactive and Inflammatory Mediators in the Promotion of Macular Edema Associated with Epiretinal Membranes. *Sci Rep.* 2017;7(1):10608.

10. Cao X, Sanchez JC, Dinabandhu A, Guo C, Patel TP, Yang Z, et al. Aqueous proteins help predict the response of patients with neovascular age-related macular degeneration to anti-VEGF therapy. *J Clin Invest.* 2022;132(2).

11. Montaner S, Sodhi A, Molinolo A, Bugge TH, Sawai ET, He Y, et al. Endothelial infection with KSHV genes in vivo reveals that vGPCR initiates Kaposi's sarcomagenesis and can promote the tumorigenic potential of viral latent genes. *Cancer cell.* 2003;3(1):23-36.

12. Rodrigues M, Kashiwabuchi F, Deshpande M, Jee K, Goldberg MF, Lutty G, et al. Expression Pattern of HIF-1alpha and VEGF Supports Circumferential Application of Scatter Laser for Proliferative Sickle Retinopathy. *Invest Ophthalmol Vis Sci.* 2016;57(15):6739-46.

Supplementary Figure Legends

Supplementary Figure 1. mRNA expression of HIF-regulated vasoactive mediators in streptozotocin (STZ)-induced diabetic mice.

(**A** and **B**) *Vegfa* and *Angptl4* mRNA expression in STZ-diabetic mice that were hyperglycemic for 1 (**A**) or 3 (**B**) months. (**C**) *Angpt2* and *Ptprb* mRNA expression in STZ-diabetic mice that were hyperglycemic for 3 months. $n = 3$ animals per condition. Data are shown as means ± SD. Statistical analyses were performed by a 2-tailed Student's t test. $*P < 0.05$; $**P < 0.01$; $***P < 0.001$; $****P < 0.0001$; NS, not significant.

Supplementary Figure 2. Retinal toxicity test in HIF-1α $^{+/-}$ and HIF-2α $^{+/-}$ mice.

(**A** and **C**) ERG of 6-week-old HIF-1α$^{+/-}$ (**A**) or HIF-2α$^{+/-}$ (**C**) mice compared to their wild type littermates. (**B** and **D**) Representative image of hematoxylin and eosin staining of retinal sections of HIF-1α$^{+/-}$ (**B**) or HIF-2α$^{+/-}$ (**D**) mice compared to their wild type littermates. n = 5 animals for each group. GCL, ganglion cell layer; INL, inner nuclear layer; ONL, outer nuclear layer; Data are shown as means ± SEM. Statistical analyses were performed by one-way ANOVA with Bonferroni's multiple-comparison test. *P < 0.05; **P < 0.01; ***P < 0.001; ****P < 0.0001; NS, not significant. Scale bar: 200μm.

Supplementary Figure 3. 32-134D inhibits HIF and HIF-regulated gene expression in STZ mice by systemic administration.

(**A**) Western blot of HIF-1α and HIF-2α expression (**B-C**), *Vegfa* and *Angptl4* mRNA expression (**B**) and *Angpt2 and Ptprb* mRNA expression (**C**) in neurosensory retinas from STZ-diabetic mice that were hyperglycemic for 6 months prior to treatment a single IP injection with 32−134D (at 40 or 80 mg/kg) 24 hours prior to sacrifice. n = 3 animals per condition; Data are shown as means ± SD. Statistical analyses were performed by one-way ANOVA with Bonferroni's multiple-comparison test. *P < 0.05; **P < 0.01; ***P < 0.001; ****P < 0.0001; NS, not significant.

Supplementary Figure 4. Intraocular administration of a well-tolerated dose of 32-134D reduces retinal neovascularization and vascular hyper-permeability in mouse models of diabetic eye disease.

mRNA expression of HIF-regulated genes at P17 in OIR mice treated with a single intraocular injection with 32-134D (70 ng) or vehicle (DMSO) at P12.

Supplementary Figure 5. Schematic representation of the multiple targets for 32-134D in diabetic eye disease.

In diabetic eye disease, sustained hyperglycemia and/or decreased perfusion of the inner retina results in accumulation of HIF-1α and HIF-2α in hypoxic retinal cells, resulting in the secretion of angiogenic mediators that promote diabetic macular edema (DME) and proliferative diabetic retinopathy (PDR). Current therapies target one of these angiogenic mediators, VEGF. VEGF expression is also targeted by 32-134D (a), along with its downstream receptor, KDR (b). 32-134D also targets other angiogenic mediators (e.g., ANGPTL4, EPO, PDGFB) secreted by retinal Müller cells which have been implicated in PDR and DME (c). 32-134D also targets endothelial cell expression of ANGPT2 (d) and VE-PTP (e), emerging targets of new therapies for diabetic eye disease currently under investigation. 32-134D also targets other angiogenic mediators (e.g., ANGPTL4, PAI-1, VEGF, MMP-2, and PAI-1) secreted by vascular endothelial cells (f) which have been implicated in diabetic eye disease. By broadly inhibiting multiple vasoactive mediators and reducing their expression back to physiologic levels, 32-134D can safely and effectively treat both PDR and DME.

Supplemental Figure 1

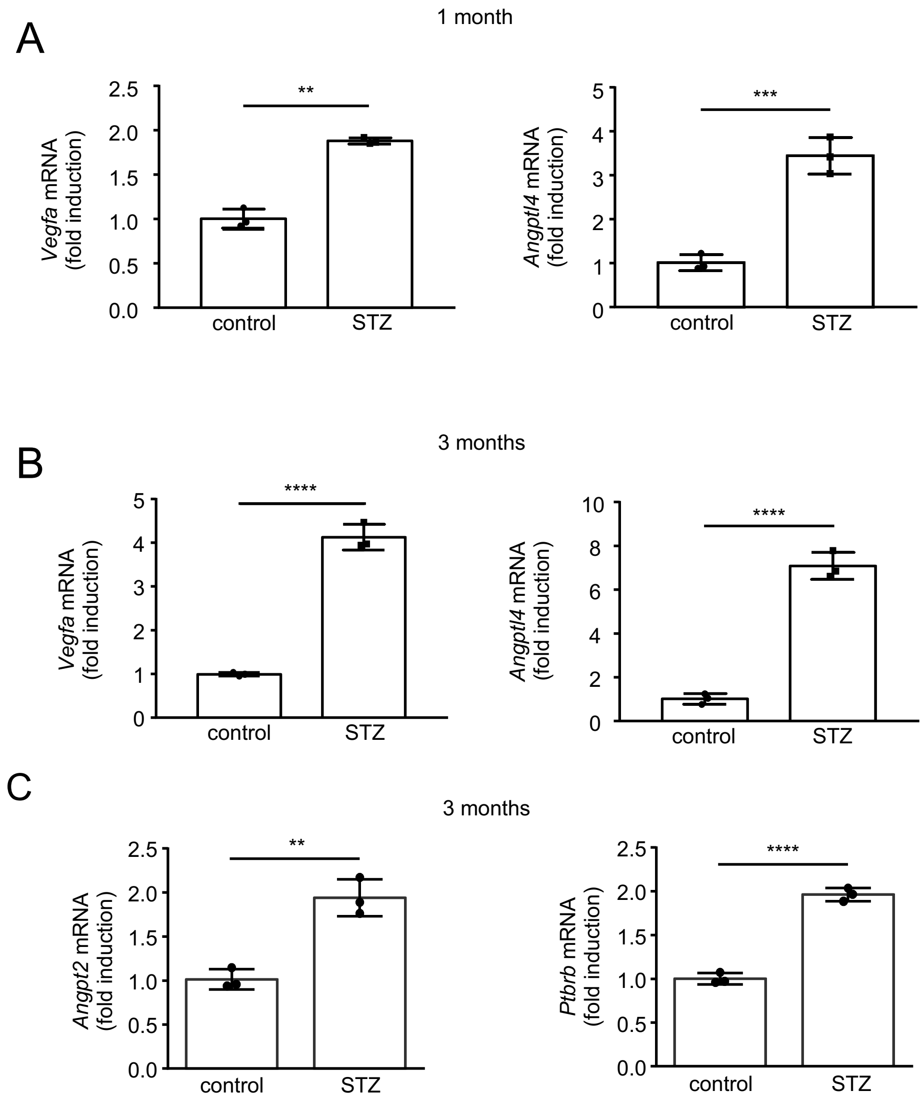

Supplemental Figure 2

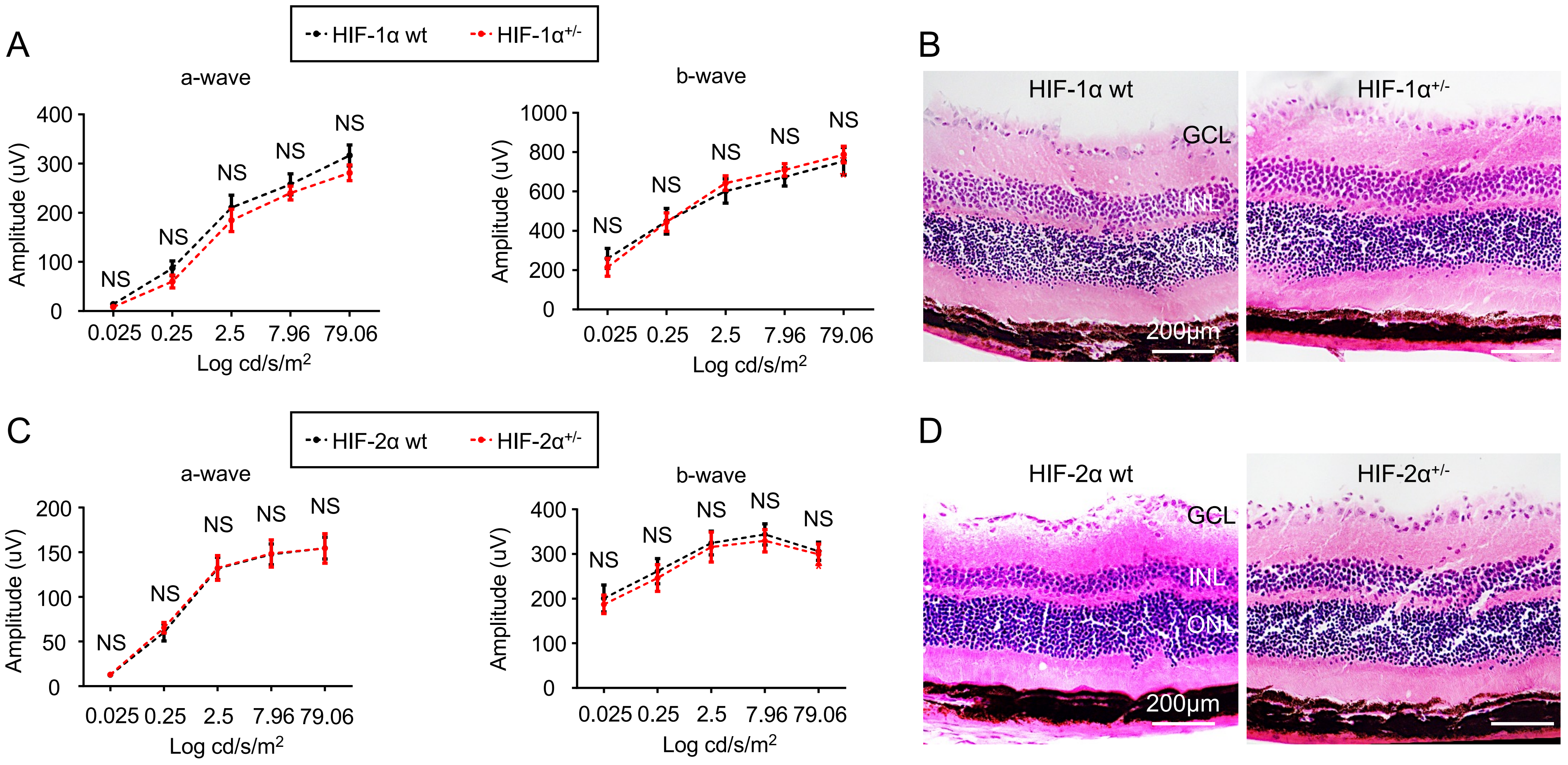

Supplemental Figure 3

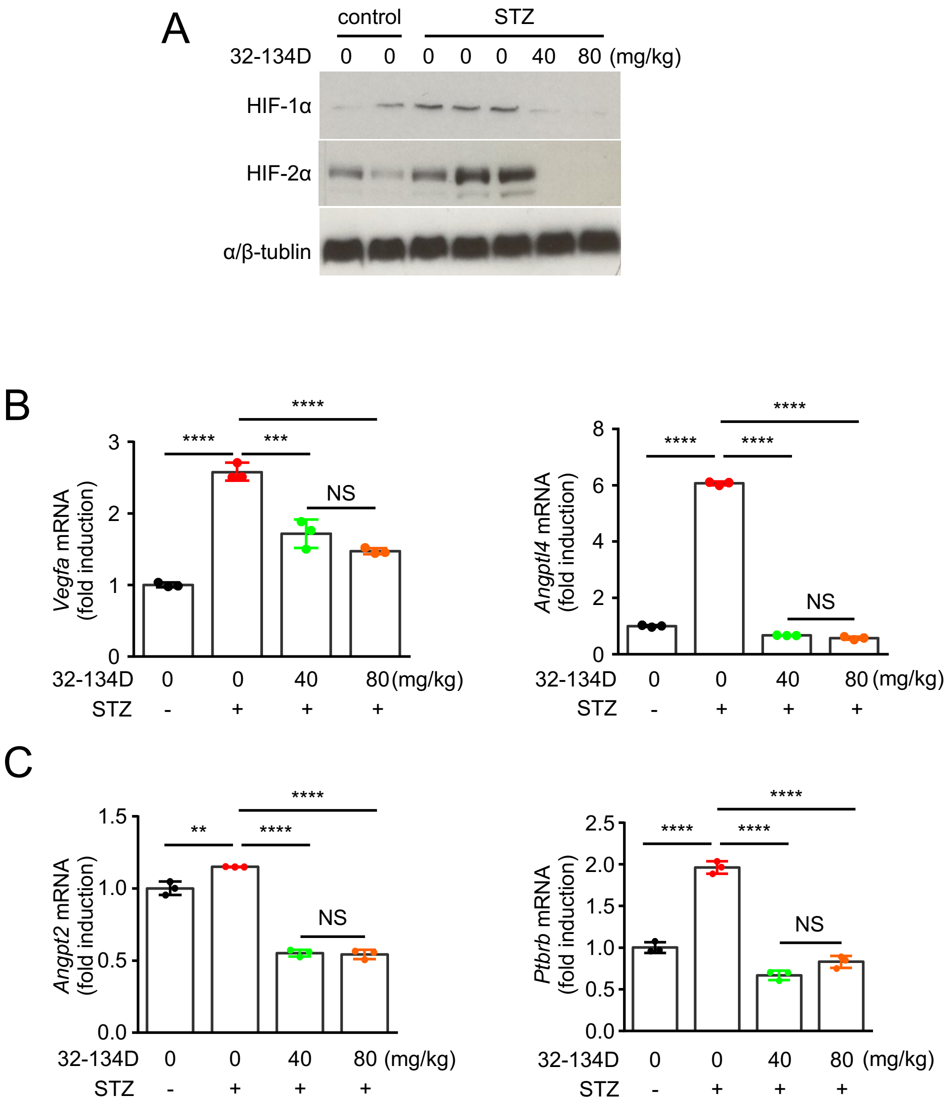

Supplemental Figure 4

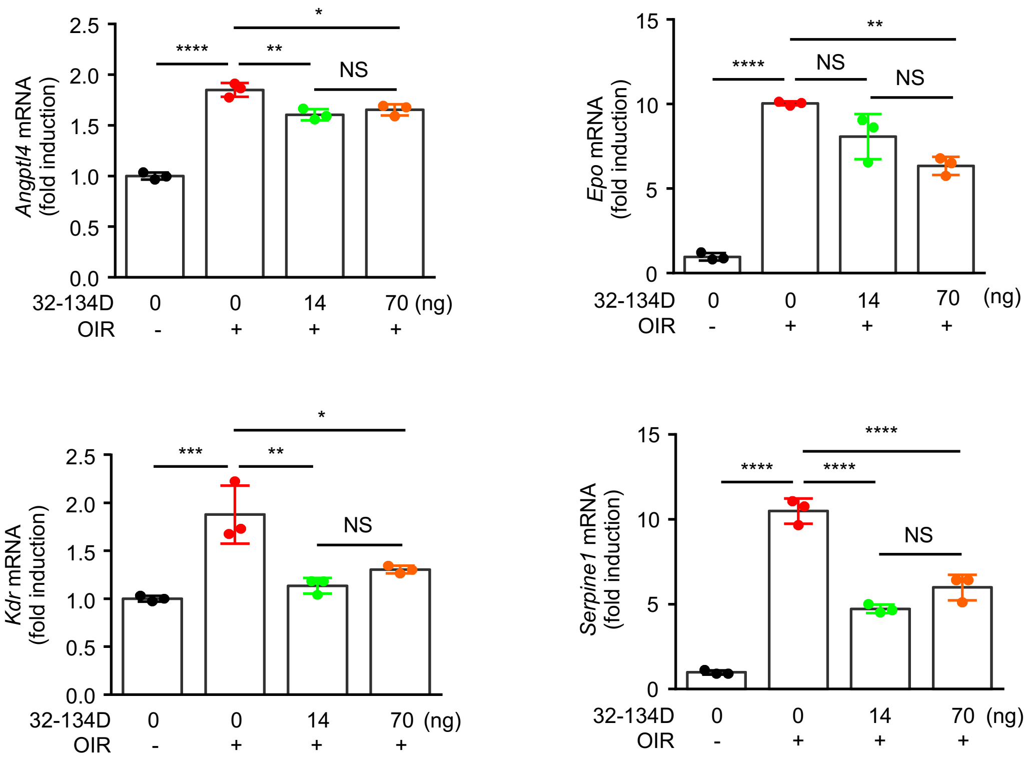

Supplemental Figure 5

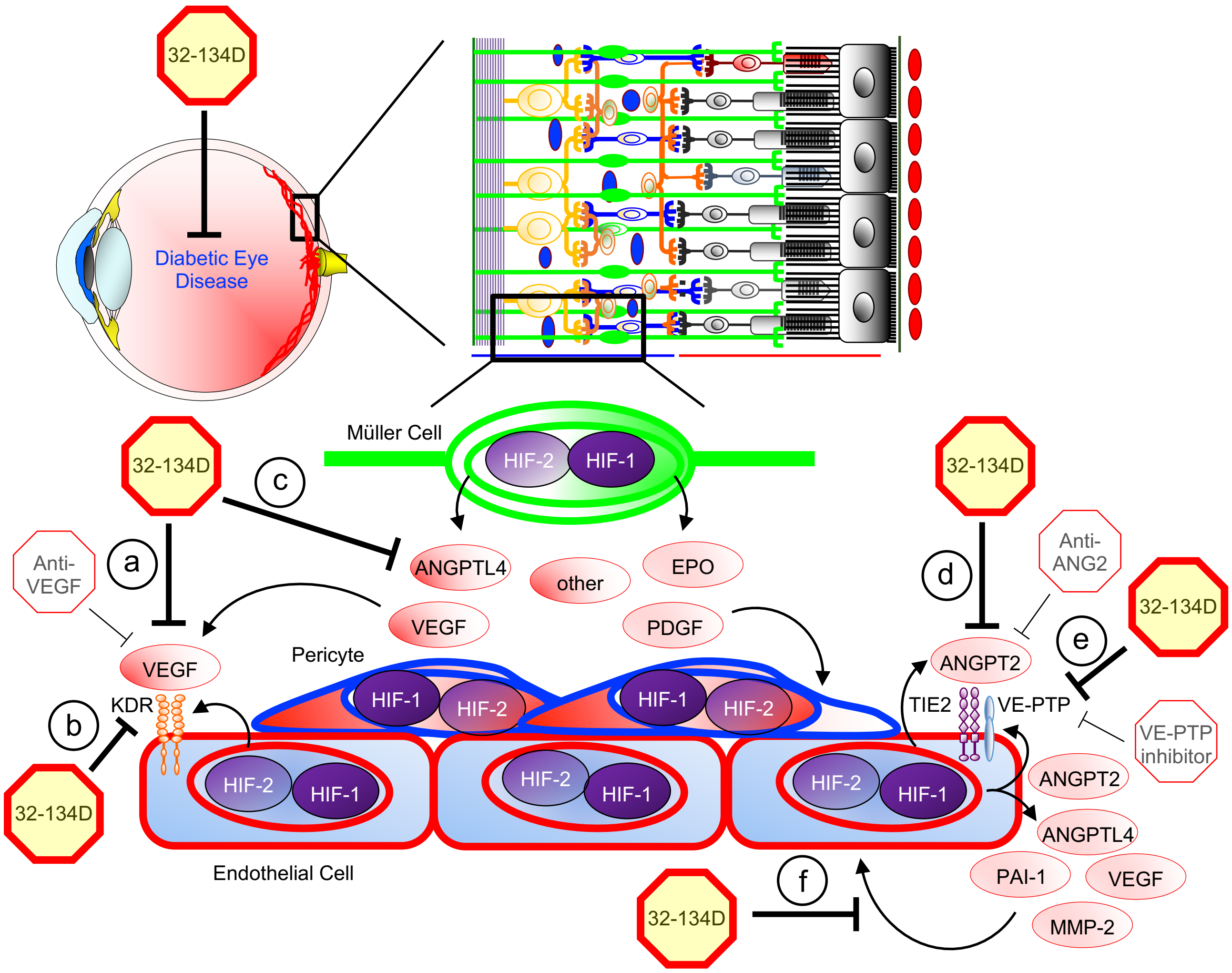

Supplement: Supplemental data [file jci-133-163290-s237.pdf]
